# Phase transition to chaos in complex ecosystems with non-reciprocal species-resource interactions

**Published:** 2024-02-27

**Authors:** Emmy Blumenthal, Jason W. Rocks, Pankaj Mehta

**Affiliations:** 1 Department of Physics, Boston University, Boston, MA 02215, USA; 2 Faculty of Computing and Data Science, Boston University, Boston, MA 02215, USA

## Abstract

Non-reciprocal interactions between microscopic constituents can profoundly shape the large-scale properties of complex systems. Here, we investigate the effects of non-reciprocity in the context of theoretical ecology by analyzing a generalization of MacArthur’s consumer-resource model with asymmetric interactions between species and resources. Using a mixture of analytic cavity calculations and numerical simulations, we show that such ecosystems generically undergo a phase transition to chaotic dynamics as the amount of non-reciprocity is increased. We analytically construct the phase diagram for this model and show that the emergence of chaos is controlled by a single quantity: the ratio of surviving species to surviving resources. We also numerically calculate the Lyapunov exponents in the chaotic phase and carefully analyze finite-size effects. Our findings show how non-reciprocal interactions can give rise to complex and unpredictable dynamical behaviors even in the simplest ecological consumer-resource models.

Many complex systems operate out of equilibrium where components generically interact non-reciprocally. Significant current research aims to untangle the implications of non-reciprocal interactions for self-organization and pattern formation. While much progress has been made towards understanding non-reciprocity in systems composed of a few types of species or fields, the consequences of non-reciprocity in more complex systems composed of many interacting components are less clear and presents interesting questions in studies of ecosystems, pattern formation, active matter, mechanical networks, and neural networks [1–5].

Large, diverse ecosystems with many types of species and resources provide a natural setting for exploring this open problem. Over the last decade, researchers have adapted methods from the statistical physics of disordered systems (e.g., replicas, the cavity method, random matrix theory) to analyze such ecosystems [6–15]. Much of this work has focused on systems with reciprocal interactions in which dynamics are often implicitly governed by an optimization function and reach a fixed point [16–19].

One notable exception are recent studies of the random Generalized Lotka–Volterra (GLV) model in which species interact non-reciprocally [20–25]. These systems can exhibit novel behaviors such as dynamic fluctuations and chaos, including unpredictable “boom-and-bust” dynamics where low-abundance species suddenly bloom to high abundance [26]. These observations suggest that non-reciprocal interactions can qualitatively change ecological dynamics in species-only models. However, the generalization of these observations to more complex ecosystems with multiple trophic layers or environmentally-mediated interactions remains unexplored.

Here, we introduce a generalization of the classic MacArthur Consumer Resource Model (MCRM) that includes non-reciprocal interactions between species and resources. Consumer-resource models, first introduced by MacArthur and Levins [19, 27, 28], have played a foundational role in modern theoretical ecology and undergird many powerful theoretical frameworks for understanding ecological competition, including contemporary niche theory and Tilman’s R* principle [29, 30].

## Theoretical setup.

**FIG. 1.**
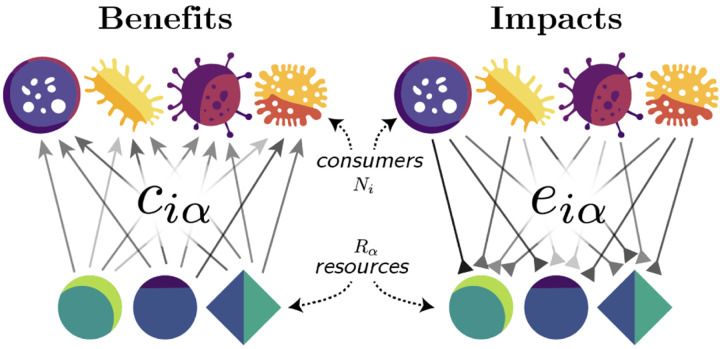
Schematic of the asymmetric MacArthur Consumer Resource Model (aMCRM). Species *i* benefits with relative weight *c*_*iα*_ from consuming resource *α* and impacts the abundance of the resource with relative weight *e*_*iα*_.

We consider an ecosystem with *i* = 1,…*, S* species which may consume *α* = 1,…*,M* distinct self-replenishing resources with dynamics governed by the equations,

(1) $$\frac{\text{d}N_{i}}{\text{d}t}=N_{i}\left(\sum_{\alpha =1}^{M}c_{i\alpha }R_{\alpha }-m_{i}\right),$$

(2) $$\frac{\text{d}R_{\alpha }}{\text{d}t}=R_{\alpha }\left(K_{\alpha }-R_{\alpha }\right)-\sum_{i=1}^{S}N_{i}e_{i\alpha }R_{\alpha },$$

where *N*_*i*_ is the population size of species *i*, *R*_*α*_ is the abundance of resource *α*, *c*_*iα*_ is the relative consumption preference of species *i* for resource *α*, *e*_*iα*_ describes the impact of species *i* on resource *α*, *m*_*i*_ is the natural mortality rate of species *i*, and *K*_*α*_ is the carrying capacity of resource *α* in the absence of consumption. We call this model the asymmetric MacArthur Consumer Resource Model (aMCRM) with a schematic provided in Fig. 1. When *e*_*iα*_ = *c*_*iα*_ the species-resource interactions become reciprocal, or symmetric, and the aMCRM reduces to the classical MacArthur Consumer Resource Model (MCRM).

To develop intuition for the role of non-reciprocity in the aMCRM, we consider the limit where the resource dynamics are fast and the resource abundances become entrained to species dynamics. In this case, we take the RHS of Eq. (2) to be zero and solve to find $R_{\alpha }=\max\left\{0,K_{\alpha }-\sum_{i}N_{i}e_{i\alpha }R_{\alpha }\right\}$ Substituting this result into the equation for species dynamics yields an effective Generalized Lotka–Volterra (GLV) equation,

(3) $$\frac{\text{d}N_{i}}{\text{d}t}=N_{i}\left(\kappa_{i}-\sum_{j=1}^{S}A_{ij}N_{j}\right),\kappa_{i}=\sum_{\alpha =1}^{M}c_{i\alpha }K_{\alpha }-m_{i},A_{ij}=\sum_{\alpha =1}^{M}c_{i\alpha }e_{j\alpha }\Theta \left(R_{\alpha }\right),$$

where *κ*_*i*_ is the effective carrying capacity for species *i* and *A*_*ij*_ is the effective species-species interaction matrix, encoding how species *j* impacts species *i* (Θ is the Heaviside function). Although typically not quantitatively accurate, this approximation provides useful qualitative insight into the nature of the non-reciprocal interactions.

In MacArthur’s original consumer-resource model, impacts and benefits are identical, *e*_*iα*_ = *c*_*iα*_. In this case, *A*_*ij*_ is symmetric, all interactions are reciprocal, the ecosystem has a unique fixed point, and the resulting steady state can be derived using an optimization principle [16]. Such behavior is expected because choosing *c*_*iα*_ = *e*_*iα*_ implicitly assumes that each species consumes resources proportional to the marginal utility conferred to that species (in the context of game theory and microeconomics, this is a “rational strategy”). When the resource-species interactions are nonreciprocal, *e*_*iα*_ ≠ *c*_*iα*_, *A*_*ij*_ is no longer symmetric, the resulting dynamics can no longer be described using an optimization principle, and there is no guarantee that the dynamics will reach a stable fixed point.

Numerical integration of the aMCRM is performed with a small immigration rate to numerically regularize simulations and ensure that when a steady state is reached, it is uninvadable (see SI section D2 for details).

## Thermodynamic limit.

To investigate the aMCRM, we work in the thermodynamic limit where the numbers of species *S* and resources *M* become very large while their ratio *M/S* is held fixed. We assume that parameters are drawn randomly from a fixed distribution analogous to quenched disorder. To ensure a proper thermodynamic limit, parameters are drawn as follows:

$$K_{\alpha }=K+\sigma_{K}\delta K_{\alpha },\;\;m_{i}=m+\sigma_{m}\delta m_{i},c_{i\alpha }=\frac{\mu_{c}}{M}+\frac{\sigma_{c}}{\sqrt{M}}d_{i\alpha },e_{i\alpha }=\frac{\mu_{e}}{M}+\frac{\sigma_{e}}{\sqrt{M}}\left(\rho d_{i\alpha }+\sqrt{1-\rho^{2}}x_{i\alpha }\right)$$

where *δK*_*α*_*, δm*_*i*_*, d*_*iα*_*, x*_*iα*_ are independent standard random variables (i.e., zero mean and unit variance) and |*ρ*| ≤ 1 is the interaction reciprocity parameter. For simplicity, we take *μ*_*c*_ = *μ*_*e*_ ≡ *μ* and *σ*_*c*_ = *σ*_*e*_ ≡ *σ* in all figures and simulations. The central limit theorem ensures that, in the thermodynamic limit, our results are agnostic to the exact form of the underlying distributions and depend only on first and second moments. Therefore, we sample all parameters from normal distributions unless otherwise specified.

With this parameterization, *ρ* controls the level of reciprocity of species-resource interactions through the correlation of consumption benefits and impacts:

(5) $$\text{corr}\left(c_{i\alpha },e_{j\beta }\right)=\rho \delta_{ij}\delta_{\alpha \beta }.$$

When *ρ* = 1, the aMCRM reduces to the fully symmetric MCRM; when *ρ* = 0, the aMCRM models completely non-reciprocal species-resource interactions. By tuning *ρ*, we can systematically explore the effects of non-reciprocity.

## Cavity method.

Just as in the original MCRM, we can analytically calculate the thermodynamic-limit behavior using the cavity method [13, 14, 31, 32]. Unlike replicas, the cavity method does not require the existence of an energy function and therefore can be extended to the aMCRM. We assume dynamics are self-averaging and described by a replica-symmetric ansatz. Using this ansatz, we derive self-consistent mean-field equations for the fraction of surviving species, the fraction of non-depleted resources, the first and second moments of the steady-state species and resource abundances, and the average linear-order responses of a resource’s abundance to a small change in its own carrying capacity and of a species’ population to a small change in its own natural mortality rate (see SI section A for detailed derivations). As seen in Figs. A6 and A8, numerical simulations and analytical predictions agree remarkably well for moderate non-reciprocity.

## Transition to dynamic phase.

**FIG. 2.**
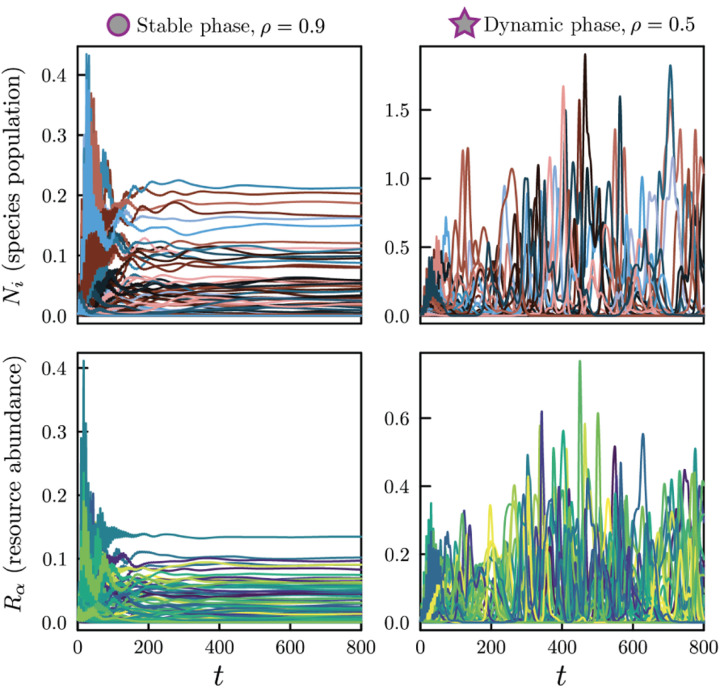
Example dynamics of the aMCRM in a community of *S* = *M* = 256 species and resources. Left: dynamics in the stable phase; species-resource interactions are nearly reciprocal. Right: dynamics in the dynamic phase; species-resource interactions are less reciprocal. The parameter values for the stable-phase and dynamic-phase simulations are respectively marked with a circle and star in Fig. 3(a).

Without reciprocal interactions, the aMCRM has no guarantee of reaching a steady state. We find that when the interaction reciprocity *ρ* is less than a critical *ρ*^⋆^, the aMCRM exhibits a phase transition from a unique self-averaging steady state to a chaotic dynamic phase. Fig. 2 shows numerical simulations of typical resource and species dynamics observed in each phase (see SI section D for simulation details [33–38]).

Using the cavity method, we can analytically compute the phase boundary between the stable and dynamic phases [31]. We perturb the nonzero steady-state species and resource abundances,$N_{i}\to N_{i}+\varepsilon \eta_{i}^{\left(N\right)}$ and $R_{\alpha }\to R_{\alpha }+\varepsilon \eta_{\alpha }^{\left(R\right)}$, where *ε* is a small parameter and $\eta_{i}^{\left(N\right)},\eta_{\alpha }^{\left(R\right)}$ are independent standard random variables, and calculate the susceptibilities d*N*_*i*_/d*ε*, d*R*_*α*_/d*ε*. Because of the disordered nature of the perturbation, the expectations of the first moments of the susceptibilities are zero, but the second moments, ⟨(d*N*_*i*_/d*ε*)^2^⟩, ⟨(d*R*_*α*_/d*ε*)^2^⟩, are nonzero (see SI section B for details).

**FIG. 3.**
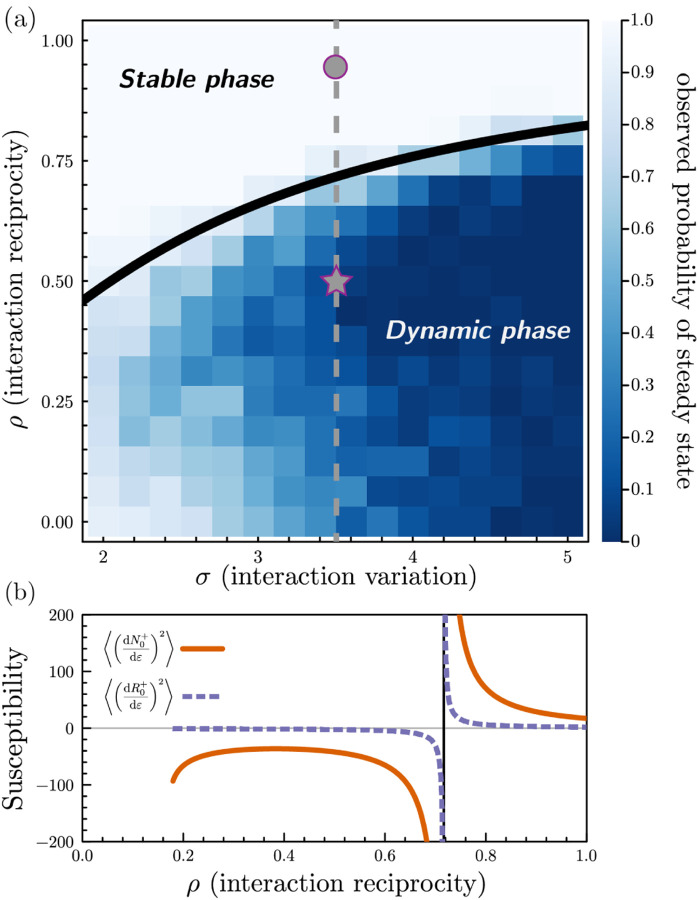
Phase diagram of the aMCRM and diverging susceptibility. (a) Heatmap of the fraction of simulations which reached steady state in finite simulation time for various values of *ρ*, the level of reciprocity of species-resource interactions, and *σ*, the magnitude of fluctuations in species-resource interactions. Overlain is the cavity method-calculated phase boundary. (b) Variances of susceptibilities of mean-field species and resources as a function of *ρ*, with *σ* fixed at the value indicated by the dashed line in (a).

The phase transition to the dynamic phase is signaled by the divergence of the these susceptibilities’ second moments (see Fig. 3). Surprisingly, we find that *ρ*^⋆^, the critical value marking the phase transition to chaos, depends on model parameters only through the species-packing fraction, the ratio of surviving species to non-depleted resources, via the expression (see SI section B):

(6) $$\rho^{⋆}=\sqrt{\frac{\#\text{of}\;\text{surviving}\;\text{species}}{\#\text{of}\;\text{non-depleted}\;\text{resources}}}.$$

When *ρ < ρ*^⋆^ the ecosystem undergoes a phase transition to chaos. As the number of surviving species and non-depleted resources are fixed by model parameters, the above equation defines a co-dimension-one phase boundary in the parameter space. Beyond this boundary in the dynamic phase, the second moments of the susceptibilities become negative, indicating that the replica-symmetric ansatz no longer holds, and its results are unstable to any perturbation.

Fig. 3(a) shows a phase diagram overlain on a heatmap of the fraction of simulations that reach steady state within a chosen finite runtime. We highlight the locations of the simulations in the stable and dynamic phases in Fig. 2 with a circle and a star, respectively. In Fig. 3(b), we plot the second moments of the susceptibilities as a function of *ρ* with fixed *σ* along the slice of phase space indicated by the dashed line in Fig. 3(a). The susceptibilities’ variances diverge at the phase transition and become invalidly negative in the dynamic phase. As the phase transition is approached, the fraction of simulations that reach steady state in a finite simulation time sharply decreases. An alternative phase diagram with parameters drawn from uniform distributions is shown in Fig. F19.

Finally, we note that for certain choices of parameters, the replica-symmetric self-consistent equations do not have a solution. This transition to infeasibility has an interesting interpretation but is not physically realized because it occurs within the dynamic phase where the replica-symmetric solution is unstable (see SI section A5). When *ρ* = 1, the instability transition and transition to cavity infeasibility coincide, and neither transition is ever achieved because in the MCRM, the competitive exclusion principle applies, keeping the right-hand side of Eq. (6) less than or equal to one. When *ρ* = 0, the system is beyond the instability transition, and the cavity infeasibility transition is achieved, meaning no replica-symmetric solution exists. Mathematically, for *ρ* = 0, the only solution to the mean-field equations is the trivial solution where all species are extinct.

## Chaos.

**FIG. 4.**
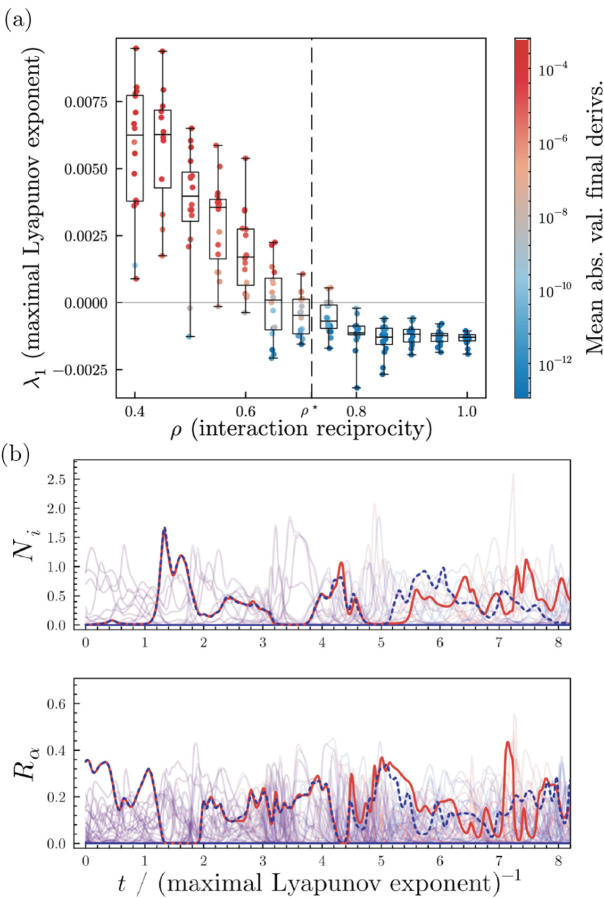
Chaos in the dynamic phase of the aMCRM. (a) Dot and box-and-whisker plot of *λ*_1_s, maximal Lyapunov exponents, for simulations at various values of *ρ*, colored by the mean of absolute values of derivatives of all species and resources at the end of the simulation which is an indicator of whether the simulation has reached steady state. (b) Two trajectories (red and blue) with slightly different initial conditions in the dynamic phase of the aMCRM. A species and a resource are highlighted to emphasize the chaotic dynamics; all other species and resources are shown at low opacity for clarity. The units of time are given by the inverse of the maximal Lyapunov exponent,$\lambda_{1}^{-1}=190$.

In order to better understand the transition to chaos, we numerically computed the maximal Lyapunov exponent *λ*_1_ of the aMCRM in the dynamic and stable phases using the “H2” method of Geist [39–42]. The maximal Lyapunov exponent characterizes how quickly trajectories from nearby initial conditions diverge (positive exponent) or converge (negative exponent). As seen in Fig. 4(a), typically, in the dynamic phase, *λ*_1_ > 0, while in the stable phase, *λ*_1_ < 0. For the parameters used in Fig. 2, |*λ*_1_| ≈ 5 × 10^−3^, indicating that the divergence or convergence of nearby trajectories occurs on a timescale of $\lambda_{1}^{-1}\approx 2\times 10^{2}$ time units. We further confirmed the existence of chaos by analyzing the generalized alignment index (GALI) which measures how a volume element formed by tangent vectors to a trajectory changes over time [41–43] (see Fig. C13). Additionally, we estimated and analyzed the Kaplan–Yorke dimension and found that it is less than the number of surviving species and resources [44]. Further details are given in SI section C.

A direct signature of chaotic dynamics is high sensitivity to initial conditions as observed in Fig. 4(b). The red and blue lines show the simulated trajectory of a single species (top) and resource (bottom) started from initial conditions with slight differences. Initially, the trajectories are almost identical before diverging from each other significantly after a few Lyapunov times.

## Finite-size effects.

Like most phase transitions, the transition between the stable and dynamic phases is a thermodynamic-limit phenomenon. In small ecosystems, the aMCRM may approach steady state even when in the dynamic phase due to finite-size effects. As a result, it is not clear in Fig. 3 what the true probability of steady state is in the thermodynamic limit. In SI section E, we quantify these effects by performing a numerical analysis to extrapolate the steady-state probabilities to infinite system size for each of the two points highlighted in Fig. 3. For both sets of parameters, we measure the distribution of steady-state times for many simulations for a variety of system sizes. Using a custom method based on maximum-likelihood estimation, we then perform a finite-size scaling collapse on these distributions, allowing us to approximately determine the steady-state probabilities as a function of system size. Our scaling collapses provide strong evidence that the probability of reaching steady state in the thermodynamic limit approaches exactly zero in the dynamic phase and one in the stable phase.

## Discussion.

In this letter, we analyzed the effects of non-reciprocal species-resource interactions on the stability of ecosystems. We introduced the asymmetric MacArthur Consumer Resource Model (aMCRM), a generalization of the MacArthur Consumer Resource Model (MCRM). Using the cavity method, we identified a phase transition between a stable phase in which a unique, uninvadable, self-averaging steady state exists and a dynamic phase with chaotic fluctuations. Remarkably, the phase boundary depends on model parameters only through the species-packing ratio—the ratio of surviving species to non-depleted resources.

Tilman analyzed stability in a two-species, two-resource system where the yields of the species on resources differs from their growth and determined “the equilibrium point will be stable if each species consumes proportionately more of the resource that more limits its own growth” [29]. The divergence between *c*_*iα*_ and *e*_*iα*_ in the aMCRM is analogous to the divergence between the yield and growth rate in Tilman’s analysis. Our results suggest that this principle generalizes to ecosystems with many species and resources; however, our analysis takes a statistical approach and uses a different model of dynamics.

We found that the chaotic regime is generic and occurs robustly and shares features with GLV models with asymmetric interactions where chaos can also be found [9, 20]. In consumer-resource models, chaotic dynamics generically occurs when the systems are well below the competitive exclusion bound, while the dynamics in GLV systems can violate the competitive exclusion principle. Unlike previous work on dynamical fluctuations in consumer-resource models [45–49], the aMCRM does not require the introduction of explicit species-species interactions to exhibit chaotic dynamics, chaos occurs below the competitive exclusion bound, the resource carrying capacities are static, the dynamics are continuous and not discrete, and the onset of chaos requires no fine-tuning and is analyzed in high dimensions. Additionally, our analysis works explicitly with the consumer-resource model and not the effective GLV model.

Collectively, these works suggest that non-reciprocal interactions can lead to complex, chaotic dynamics in systems with many types of species/fields. In particular, like GLV models, we also find that species and resources often jump rapidly between low and high abundances. In the future, it will be interesting to see if the methods developed in Ref. 26 in the context of GLV systems generalize to explain boom-and-bust dynamics in consumer-resource models and derive relevant correlation functions and dynamical susceptibilities. Preliminary results suggest that other consumer resource models with non-reciprocal species-resource interactions, such as that with externally supplied resources [50], also exhibit chaotic dynamics; we hope to explore this in future work. Finally, further investigations may seek to understand these phenomena in the context of ecological processes such as immigration, alternative resource dynamics [16, 50], the addition of network and metabolic structure into interactions [51–53], the inclusion of additional trophic structure [54], and spatial and temporal structure [55].

## Supplementary Material

1
