# Supplementary material for: Phase transition to chaos in complex ecosystems with non-reciprocal species-resource interactions
Source: ArXiv. 2024 Feb 27:arXiv:2308.15757v2. Originally published 2023 Aug 30. Preprint. [Version 2] (PMC10491343)
Supplement: 1 [file NIHPP2308.15757v2-supplement-1.pdf]

CONTENTS

References 5

A. Cavity calculation 9
1. Setup 9
2. Cavity solution 10
a. Self-consistency equations for species populations 10
b. Self-consistency equations for resource abundances 12
3. ReLU function-transformed normal distributions 13
4. Final self-consistency equations 14
5. Infeasibility of the cavity solution 15
6. Comparison of cavity and simulation results 16
7. Cavity susceptibilities 16

B. Stability phase transition in the thermodynamic limit 18

C. Chaotic nature of dynamics 21
1. Analysis of the Lyapunov exponents 22
2. Analysis of the generalized alignment index (GALI) 23
3. Analysis of effective dimension of dynamics 24

D. Simulation and numerical methods 25
1. Numerical integration 25
2. Surviving/depletion cutoffs, numerical instability, and small immigration 25
3. Numerically solving the cavity self-consistency equations and calculating phase boundaries 25

E. Finite size scaling analysis 27
1. Modeling simulation outcomes 27
2. Likelihood function 28
3. Fitting the model 28
4. Scaling of parameters with system size and discussion 29

F. Additional simulations 31

G. Parameters in figures 32
Figure 1 (schematic) details 32
Figure 2 (example dynamics) simulation parameters 32
Figure 3 (phase diagram) simulation parameters 32
Figure 4 (Lyapunov dot plot and diverging trajectories) simulations details 32

Appendix A: Cavity calculation

The objective of the cavity calculation is to find the steady-state behavior of the aMCRM. In particular, we will find the distribution of the steady-state abundances of the species and resources. The cavity method takes advantage of the system's self-averaging behavior. A system is said to have self-averaging behavior if the distribution of properties of constituents are independent of the exact realization of quenched disorder. This means that constituents' properties can be treated as random variates drawn from a distribution that is common to all systems with the same distribution of quenched disorder. A consequence of this is that an average of some observable taken over constituents given a fixed realization of quenched disorder is equal to the average of the observable for one constituent taken over all realizations of quenched disorder. The emergence of this self-averaging behavior is a consequence of the central limit theorem and quenched disorder, as we will see. In Fig. A5, we compare the distribution of steady-state resource and species abundance for a single realization of quenched disorder to the distribution of abundances predicted by the cavity method.

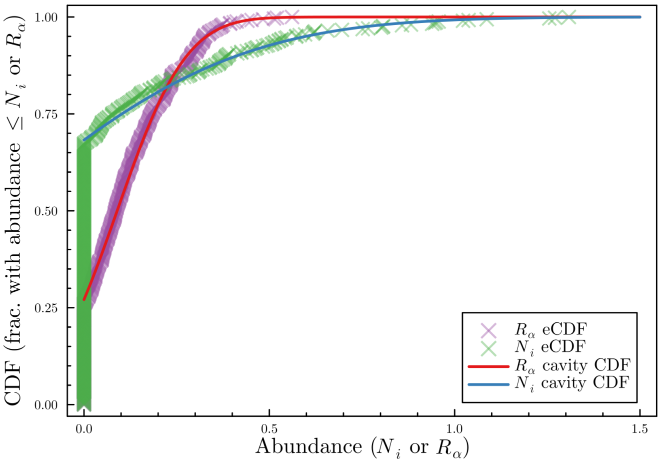

FIG. A5. Comparison of the empirical cumulative distribution functions (eCDFs) of the steady-state abundances of species ($S = 512$) and resources ($M = 512$) for a single realization of quenched disorder and the distribution predicted by the cavity calculation; simulations are performed in the stable phase. See appendix G for simulation parameters.

1. Setup

We begin by introducing the constituent averages,

$$\langle R \rangle \equiv \frac{1}{M}\sum_{\alpha=1}^{M} \overline{R}_\alpha, \qquad \langle N \rangle \equiv \frac{1}{S}\sum_{i=1}^{S} \overline{N}_i, \tag{A1}$$

where we have introduced the notation $\overline{X}$ to denote the steady-state value of a quantity X. With these quantities defined, we can write the steady-state aMCRM as,

$$0 = \frac{\mathrm{d}N_i}{\mathrm{d}t} = \overline{N}_i\left[g + \frac{\sigma_c}{\sqrt{M}}\sum_{\alpha=1}^{M} d_{i\alpha}\overline{R}_\alpha - \sigma_m \delta m_i\right], \tag{A2}$$

$$0 = \frac{\mathrm{d}R_\alpha}{\mathrm{d}t} = \overline{R}_\alpha\left[\kappa - \overline{R}_\alpha - \frac{\sigma_e}{\sqrt{M}}\sum_{i=1}^{S} N_i\left(\rho d_{i\alpha} + \sqrt{1-\rho^2}x_{i\alpha}\right) + \sigma_K \delta K_\alpha\right], \tag{A3}$$

where

$$g \equiv \mu_c \langle R \rangle - m, \qquad \kappa \equiv K - \mu_e \gamma^{-1}\langle N \rangle, \qquad \gamma \equiv \frac{M}{S}. \tag{A4}$$

2. Cavity solution

The cavity method begins by introducing a new species $i = 0$ and a new resource $\alpha = 0$. We will later treat the new terms introduced by these variables as small perturbations. With the new species and resource, we can write the steady-state aMCRM for the existing species $i = 1, \ldots, S$ and resources $\alpha = 1, \ldots, M$ as,

$$0 = \frac{\mathrm{d}N_i}{\mathrm{d}t} = \overline{N}_i \left[\mu_c \langle R \rangle - \left(m - \frac{\sigma_c}{\sqrt{M}} d_{i0} R_0 \right) + \frac{\sigma_c}{\sqrt{M}} \sum_{\alpha=1}^{M} d_{i\alpha} \overline{R}_\alpha - \sigma_m \delta m_i \right], \tag{A5}$$

$$0 = \frac{\mathrm{d}R_\alpha}{\mathrm{d}t} = \overline{R}_\alpha \Bigg[\left(K - \frac{\sigma_e}{\sqrt{M}} N_0 \Big(\rho d_{0\alpha} + \sqrt{1-\rho^2} x_{0\alpha} \Big) \right) - \mu_e \gamma^{-1} \langle N \rangle - \overline{R}_\alpha$$
$$- \frac{\sigma_e}{\sqrt{M}} \sum_{i=1}^{S} \overline{N}_i \Big(\rho d_{i\alpha} + \sqrt{1-\rho^2} x_{i\alpha} \Big) + \sigma_K \delta K_\alpha \Bigg]. \tag{A6}$$

For the new species and resource, the steady-state aMCRM says,

$$0 = \frac{\mathrm{d}N_0}{\mathrm{d}t} = \overline{N}_0 \left[g + \frac{\sigma_c}{\sqrt{M}} \sum_{\alpha=1}^{M} d_{0\alpha} \overline{R}_\alpha + M^{-1/2} \sigma_c d_{00} \overline{R}_0 - \sigma_m \delta m_0 \right], \tag{A7}$$

$$0 = \frac{\mathrm{d}R_0}{\mathrm{d}t} = \overline{R}_0 \Bigg[\kappa - R_0 - \frac{\sigma_e}{\sqrt{M}} \sum_{i=1}^{S} \overline{N}_j \left(\rho d_{i0} + \sqrt{1-\rho^2} x_{i0} \right)$$
$$- \frac{\sigma_e}{\sqrt{M}} \overline{N}_0 \left(\rho d_{00} + \sqrt{1-\rho^2} x_{00} \right) + \sigma_K \delta K_\alpha \Bigg]. \tag{A8}$$

Next, we will analyze the perturbed system relative to the unperturbed system. A quantity with \0 represents the value before adding the new variables to the system. Looking to Eqs. A5 and A6, we see that the presence of the new species and resource effectively perturbs the model parameters as,

$$m_i \to m_i - \frac{\sigma_c}{\sqrt{M}} d_{i0} \overline{R}_0, \qquad K_\alpha \to K_\alpha - \frac{\sigma_e}{\sqrt{M}} \overline{N}_0 \left(\rho d_{0\alpha} + \sqrt{1-\rho^2} x_{0\alpha} \right). \tag{A9}$$

In the thermodynamic limit where M and S are large, we model the perturbation using linear response:

$$\overline{N}_i = \overline{N}_{i\backslash 0} - \frac{\sigma_e}{\sqrt{M}} \sum_{\beta=1}^{M} \chi^{(N)}_{i\beta} \left(\rho d_{0\beta} + \sqrt{1-\rho^2} x_{0\beta} \right) \overline{N}_0 - \frac{\sigma_c}{\sqrt{M}} \sum_{j=1}^{S} \nu^{(N)}_{ij} d_{j0} \overline{R}_0, \tag{A10}$$

$$\overline{R}_\alpha = \overline{R}_{\alpha\backslash 0} - \frac{\sigma_e}{\sqrt{M}} \sum_{\beta=1}^{M} \chi^{(R)}_{\alpha\beta} \left(\rho d_{0\beta} + \sqrt{1-\rho^2} x_{0\beta} \right) \overline{N}_0 - \frac{\sigma_c}{\sqrt{M}} \sum_{j=1}^{S} \nu^{(R)}_{\alpha j} d_{j0} \overline{R}_0, \tag{A11}$$

where we have defined the susceptibility matrices:

$$\chi^{(N)}_{i\beta} \equiv \frac{\partial \overline{N}_i}{\partial K_\beta}, \qquad \chi^{(R)}_{\alpha\beta} \equiv \frac{\partial \overline{R}_\alpha}{\partial K_\beta}, \tag{A12}$$

$$\nu^{(N)}_{ij} \equiv \frac{\partial \overline{N}_i}{\partial m_j}, \qquad \nu^{(R)}_{\alpha j} \equiv \frac{\partial \overline{R}_\alpha}{\partial m_j}. \tag{A13}$$

a. *Self-consistency equations for species populations*

With the perturbation introduced and susceptibilities defined, we will exploit the linear response approximation to derive the self-consistency equations for the species populations. Substituting Eqs. A10, A11 into the aMCRM

equation for the additional species [Eq. (A7)], we obtain,

$$0 = \overline{N}_0 \Bigg[g + \frac{\sigma_c}{\sqrt{M}} \sum_{\alpha=1}^{M} d_{0\alpha} \overline{R}_{\alpha\backslash 0} - \frac{\sigma_c \sigma_e}{M} \sum_{\alpha,\beta=1}^{M} \chi^{(R)}_{\alpha\beta} d_{0\alpha} \left(\rho d_{0\beta} + \sqrt{1-\rho^2} x_{0\beta} \right) \overline{N}_0 - \frac{\sigma_c^2}{M} \sum_{\alpha=1}^{M} \sum_{j=1}^{S} \nu^{(R)}_{\alpha j} d_{0\alpha} d_{j0} \overline{N}_0 + \frac{\sigma_c}{\sqrt{M}} d_{00} \overline{R}_0 - \sigma_m \delta m_0 \Bigg]. \tag{A14}$$

Taking the mean of the third term with respect to the new matrix elements $d_{0\alpha}$ and $x_{0\alpha}$, we obtain

$$\begin{aligned} &\left\langle \frac{\sigma_c \sigma_e}{M} \sum_{\alpha,\beta=1}^{M} \chi^{(R)}_{\alpha\beta} d_{0\alpha} \left(\rho d_{0\beta} + \sqrt{1-\rho^2} x_{0\beta} \right) \overline{N}_0 \right\rangle \\ &= \overline{N}_0 \frac{\sigma_c \sigma_e}{M} \sum_{\alpha,\beta=1}^{M} \chi^{(R)}_{\alpha\beta} \left(\rho \langle d_{0\alpha} d_{0\beta} \rangle + \langle d_{0\alpha} \rangle \langle x_{0\beta} \rangle \sqrt{1-\rho^2} \right) \\ &= \overline{N}_0 \frac{\sigma_c \sigma_e}{M} \sum_{\alpha,\beta=1}^{M} \chi^{(R)}_{\alpha\beta} \left(\rho \delta_{\alpha\beta} + 0 \times 0 \sqrt{1-\rho^2} \right) = \overline{N}_0 \sigma_c \sigma_e \rho \chi, \end{aligned} \tag{A15}$$

where we have used that $d_{0\alpha}$ and $x_{0\beta}$ are independent and have defined,

$$\chi \equiv \frac{1}{M} \sum_{\alpha=1}^{M} \chi^{(R)}_{\alpha\alpha}, \tag{A16}$$

to be the trace of the $\overline{R}_\alpha \leftarrow K_\beta$ susceptibility matrix divided by the number of resources. The variance of this term is $O(M^{-1})$, which can be verified by expanding its second moment. The mean of the fourth term is zero because $d_{0\alpha}$ and d_{j0} are uncorrelated when $\alpha = 1, \ldots, M,\ j = 1, \ldots, S$ and its variance is $O(M^{-1})$. Discarding terms of order $O(M^{-1/2})$ and higher, we obtain,

$$0 = \overline{N}_0 \left(g - \sigma_c \sigma_e \rho \chi \overline{N}_0 + \frac{\sigma_c}{\sqrt{M}} \sum_{\alpha=1}^{M} d_{0\alpha} \overline{R}_{\alpha\backslash 0} - \sigma_m \delta m_0 \right) + O(M^{-1/2}). \tag{A17}$$

The last two terms above are a sum of many independent random variables, so, by the central limit theorem, we can model these terms as a sum of normal random variables. The mean of these terms is,

$$\begin{aligned} \left\langle \frac{\sigma_c}{\sqrt{M}} \sum_{\alpha=1}^{M} d_{0\alpha} \overline{R}_{\alpha\backslash 0} - \sigma_m \delta m_0 \right\rangle &= \frac{\sigma_c}{\sqrt{M}} \sum_{\alpha=1}^{M} \langle d_{0\alpha} \rangle \overline{R}_{\alpha\backslash 0} - \sigma_m \langle \delta m_0 \rangle \\ &= \frac{\sigma_c}{\sqrt{M}} \sum_{\alpha=1}^{M} 0 \times \overline{R}_{\alpha\backslash 0} - \sigma_m \times 0 = 0. \end{aligned} \tag{A18}$$

The variance of these terms is,

$$\begin{aligned} \sigma_g^2 \equiv \mathrm{Var} \left[\frac{\sigma_c}{\sqrt{M}} \sum_{\alpha=1}^{M} d_{0\alpha} \overline{R}_{\alpha\backslash 0} - \sigma_m \delta m_0 \right] &= \frac{\sigma_c^2}{M} \sum_{\alpha=1}^{M} \overline{R}^2_{\alpha\backslash 0} \mathrm{Var}\left[d_{0\alpha} \right] + \sigma_m^2 \mathrm{Var}\left[\delta m_0 \right] \\ &= \frac{\sigma_c^2}{M} \sum_{\alpha=1}^{M} \overline{R}^2_{\alpha\backslash 0} + \sigma_m^2 = \sigma_c^2 q_R + \sigma_m^2, \end{aligned} \tag{A19}$$

where we have defined $q_R \equiv M^{-1} \sum_{\alpha=1}^{M} \overline{R}^2_{\alpha\backslash 0}$. Due to the self-averaging nature of the system and that the size of the perturbation is of order $O(M^{-1/2})$, we have $q_R \approx \langle \overline{R}^2_0 \rangle$. Let $Z_N \sim \mathcal{N}(0,1)$ be a unit normal random variable. The large-M limit approximate steady-state condition for the perturbing species becomes,

$$0 = \overline{N}_0 \left(g - \sigma_c \sigma_e \rho \chi \overline{N}_0 + \sigma_g Z_N \right). \tag{A20}$$

Solving for $\overline{N}_0$ and discarding invadable solutions, we obtain,

$$\boxed{\overline{N}_0 = \max\left\{0, \frac{g + \sigma_g Z_N}{\sigma_c \sigma_e \rho \chi}\right\}}. \tag{A21}$$

Additionally, observe that if $\chi \to 0$, the cavity solution diverges; this will be discussed in further detail in SI section A 5.

b. Self-consistency equations for resource abundances

Now, we repeat this process to find a self-consistency equation for the resources. We substitute the linear response approximation for species into the aMCRM steady-state equation for the additional resource:

$$0 = \overline{R}_0 \Bigg[\kappa - \overline{R}_0 - \frac{\sigma_e}{\sqrt{M}} \sum_{i=1}^{S} \overline{N}_{i\setminus 0} \left(\rho d_{i0} + \sqrt{1-\rho^2} x_{i0}\right) \tag{A22}$$
$$+ \frac{\sigma_e^2}{M} \sum_{i=1}^{S} \sum_{\beta=1}^{M} \chi_{i\beta}^{(N)} \left(\rho d_{i0} + \sqrt{1-\rho^2} x_{i0}\right) \left(\rho d_{0\beta} + \sqrt{1-\rho^2} x_{0\beta}\right) \overline{N}_0$$
$$+ \frac{\sigma_e \sigma_c}{M} \sum_{i,j=1}^{S} \nu_{ij}^{(N)} \left(\rho d_{i0} + \sqrt{1-\rho^2} x_{i0}\right) d_{j0} \overline{R}_0 - \frac{\sigma_e}{\sqrt{M}} \overline{N}_0 \left(\rho d_{00} + \sqrt{1-\rho^2} x_{00}\right) + \sigma_K \delta K_\alpha \Bigg].$$

Observe that the fourth term (involving $\chi_{i\beta}^{(N)}$) has zero mean and variance of order $O(M^{-1})$; this can be seen by recalling that $d_{i0}, d_{0\beta}, x_{i0}, x_{0\beta}$ are all independent for $i, \beta \geq 1$. Similarly, we see that the variance of the fifth term (involving $\nu_{ij}^{(N)}$) is of order $O(M^{-1})$. We will ignore fluctuations of order $O(M^{-1/2})$. The mean of the fifth term is,

$$\frac{\sigma_e \sigma_c}{M} \sum_{i,j=1}^{S} \nu_{ij}^{(N)} \left(\rho d_{i0} + \sqrt{1-\rho^2} x_{i0}\right) d_{j0} \overline{R}_0 = \overline{R}_0 \frac{\sigma_e \sigma_c}{M} \sum_{i,j=1}^{S} \nu_{ij}^{(N)} \left(\rho \langle d_{i0} d_{j0} \rangle + \sqrt{1-\rho^2} \langle x_{i0} \rangle \langle d_{j0} \rangle\right)$$
$$= \overline{R}_0 \sigma_e \sigma_c \frac{S}{M} \frac{1}{S} \sum_{i,j=1}^{S} \nu_{ij}^{(N)} \left(\rho \delta_{ij} + \sqrt{1-\rho^2} \times 0 \times 0\right) = \rho \sigma_e \sigma_c \gamma^{-1} \nu \overline{R}_0, \tag{A23}$$

where we have defined,

$$\nu \equiv \frac{1}{S} \sum_{i=1}^{S} \nu_{ii}^{(N)}, \tag{A24}$$

which is the trace of the $\overline{N}_i \leftarrow m_j$ susceptibility matrix divided by S. Discarding terms of order $O(M^{-1/2})$, we obtain,

$$0 = \overline{R}_0 \left(\kappa - \overline{R}_0 - \frac{\sigma_e}{\sqrt{M}} \sum_{i=1}^{S} \overline{N}_{i\setminus 0} \left(\rho d_{i0} + \sqrt{1-\rho^2} x_{i0}\right) + \rho \sigma_e \sigma_c \gamma^{-1} \nu \overline{R}_0 + \sigma_K \delta K_0 \right) + O(M^{-1/2}). \tag{A25}$$

Now, observe the third and last terms are a sum of many independent random variables, meaning we can apply the central limit theorem and model the sum of these terms as a normal random variable. The mean of these terms is,

$$\left\langle \sigma_K \delta K_0 - \frac{\sigma_e}{\sqrt{M}} \sum_{i=1}^{S} \overline{N}_{i\setminus 0} \left(\rho d_{i0} + \sqrt{1-\rho^2} x_{i0}\right) \right\rangle = \sigma_K \langle \delta K_0 \rangle - \frac{\sigma_e}{\sqrt{M}} \sum_{i=1}^{S} \overline{N}_{i\setminus 0} \left(\rho \langle d_{i0} \rangle + \sqrt{1-\rho^2} \langle x_{i0} \rangle\right)$$
$$= \sigma_K \times 0 - \frac{\sigma_e}{\sqrt{M}} \sum_{i=1}^{S} \overline{N}_{i\setminus 0} \left(\rho \times 0 + \sqrt{1-\rho^2} \times 0\right) = 0. \tag{A26}$$

The variance is,

$$\begin{aligned}\sigma_\kappa^2 &\equiv \mathrm{Var}\left[\sigma_K \delta K_0 - \frac{\sigma_e}{\sqrt{M}}\sum_{i=1}^{S}\overline{N}_{i\setminus 0}\left(\rho d_{i0} + \sqrt{1-\rho^2}x_{i0}\right)\right] \\ &= \sigma_K^2 \mathrm{Var}\left[\delta K_0\right] + \frac{\sigma_e^2}{M}\sum_{i=1}^{S}\mathrm{Var}\left[\overline{N}_{i\setminus 0}\left(\rho d_{i0} + \sqrt{1-\rho^2}x_{i0}\right)\right] \\ &= \sigma_K^2 + \frac{\sigma_e^2}{M}\sum_{i=1}^{S}\overline{N}_{i\setminus 0}^2\left(\rho^2 \mathrm{Var}\left[d_{i0}\right] + (1-\rho^2)\mathrm{Var}\left[x_{i0}\right]\right) = \sigma_K^2 + \sigma_e^2 \frac{S}{M}\frac{1}{S}\sum_{i=1}^{S}\overline{N}_{i\setminus 0}^2 = \sigma_K^2 + \gamma^{-1}\sigma_e^2 q_N,\end{aligned} \tag{A27}$$

where $q_N \equiv \frac{1}{S}\sum_{i=1}^{S} N_{i\setminus 0}^2$ which is approximately equal to $\langle \overline{N}_0^2 \rangle$. The approximate steady-state condition for the added resource then becomes,

$$0 = \overline{R}_0\left(\kappa - \overline{R}_0 + \sigma_\kappa Z_R + \rho\sigma_e\sigma_c\gamma^{-1}\nu\overline{R}_0\right), \tag{A28}$$

where $Z_R \sim \mathcal{N}(0,1)$ is a standard normal random variable. Solving for $\overline{R}_0$ and discarding invadable solutions gives,

$$\boxed{\overline{R}_0 = \max\left\{0, \frac{\kappa + \sigma_\kappa Z_R}{1 - \rho\sigma_e\sigma_c\gamma^{-1}\nu}\right\}}. \tag{A29}$$

3. ReLU function-transformed normal distributions

In these computations, we regularly work with normal distributions that are transformed by the 'ReLU' function: $\mathrm{ReLU}(x) = \max\{0, x\} = x\Theta(x)$. If Z is a standard normal random variable, the PDF of $\mathrm{ReLU}(\sigma Z + \mu)$ is,

$$p_{\mathrm{ReLU}(\sigma Z+\mu)}(z) = \delta(z)\Phi(-\mu/\sigma) + \frac{1}{\sqrt{2\pi}\sigma}e^{-(z-\mu)^2/2\sigma^2}\Theta(z), \tag{A30}$$

where,

$$\Phi(x) = \frac{1}{\sqrt{2\pi}}\int_{-\infty}^{x} \mathrm{d}z\, e^{-z^2/2} = \frac{1}{2}\left(1 + \mathrm{erf}(x/\sqrt{2})\right), \tag{A31}$$

is the standard normal CDF. The jth ($j \geq 1$) moment is then,

$$\begin{aligned}W_j(\mu,\sigma) &= \langle \mathrm{ReLU}(\sigma Z + \mu)^j \rangle = 0 + \frac{1}{\sqrt{2\pi}\sigma}\int_0^\infty \mathrm{d}z\, z^j e^{-(z-\mu)^2/2\sigma^2} \\ &= \sigma^j \int_{-\mu/\sigma}^{\infty} \frac{\mathrm{d}z}{\sqrt{2\pi}} e^{-z^2/2}(z + \mu/\sigma)^j, \\ &= \frac{2^{-3/2}}{\sqrt{\pi}}(\sqrt{2}\sigma)^j\left[j\frac{\mu}{\sigma}\Gamma\left(\frac{j}{2}\right)\,{}_1F_1\left(\frac{1-j}{2};\frac{3}{2};-\frac{\mu^2}{2\sigma^2}\right) + \sqrt{2}\Gamma\left(\frac{j+1}{2}\right)\,{}_1F_1\left(-\frac{j}{2};\frac{1}{2};-\frac{\mu^2}{2\sigma^2}\right)\right],\end{aligned} \tag{A32}$$

where ${}_1F_1$ is the confluent hypergeometric function of the first kind, and Γ is the gamma function. Observe that $W_j(\mu/\alpha, \sigma/\alpha) = \alpha^{-j}W_j(\mu,\sigma)$. Additionally,

$$W_0(x,1) = 1, \tag{A33}$$

$$W_1(x,1) = \frac{1}{\sqrt{2\pi}}e^{-x^2/2} + x\Phi(x), \tag{A34}$$

$$W_2(x,1) = \frac{1}{\sqrt{2\pi}}xe^{-x^2/2} + (1+x^2)\Phi(x). \tag{A35}$$

It follows from integration by parts,

$$W_2(x,1) = \Phi(x) + xW_1(x,1). \tag{A36}$$

Additionally, for a random variable $\Theta(\sigma Z+\mu)$, the PDF is,

$$p_{\Theta(\sigma Z+\mu)}(z)=\frac{1}{2}\left[1+\operatorname{erf}\left(\frac{\mu}{\sigma\sqrt{2}}\right)\right]\delta(z-1)+\frac{1}{2}\operatorname{erfc}\left(\frac{\mu}{\sigma\sqrt{2}}\right)\delta(z), \tag{A37}$$

so the jth moment ($j\geq 1$) is,

$$\langle\Theta(\sigma Z+\mu)^j\rangle=0+\frac{1}{2}\left[1+\operatorname{erf}\left(\frac{\mu}{\sigma\sqrt{2}}\right)\right]1^j=\frac{1}{2}\left[1+\operatorname{erf}\left(\frac{\mu}{\sigma\sqrt{2}}\right)\right]=\Phi(\mu/\sigma). \tag{A38}$$

4. Final self-consistency equations

Some essential quantities of interest are the expected fraction of surviving species ϕ_N and fraction of non-depleted resources ϕ_R. These quantities are computed using the moments calculated in section A 3 and Eqs. A20, A28:

$$\boxed{\phi_N=\left\langle\Theta(\overline{N}_0)\right\rangle=\Phi(\Delta_g)}, \tag{A39}$$

$$\boxed{\phi_R=\left\langle\Theta(\overline{R}_0)\right\rangle=\Phi(\Delta_\kappa)}, \tag{A40}$$

where $\Delta_g=g/\sigma_g$ and $\Delta_\kappa=\kappa/\sigma_\kappa$ and Θ is the Heaviside step function with the convention $\Theta(0)=0$. Next, we can differentiate our expressions for $\overline{N}_0$ and $\overline{R}_0$ to find,

$$\frac{\partial\overline{N}_0}{\partial m}=\frac{\partial}{\partial m}\frac{g+\sigma_g Z_N}{\sigma_c\sigma_e\rho\chi}\Theta\left(\overline{N}_0\right)=-\frac{1}{\sigma_c\sigma_e\rho\chi}\Theta(\overline{N}_0)+\left[\overline{N}_0\delta(\overline{N}_0)\text{-term}\right]$$

$$\implies\left\langle\frac{\partial\overline{N}_0}{\partial m}\right\rangle=\boxed{\nu=-\frac{\phi_N}{\sigma_c\sigma_e\rho\chi}} \tag{A41}$$

$$\frac{\partial\overline{R}_0}{\partial K}=\frac{\partial}{\partial K}\frac{\kappa+\sigma_\kappa Z_R}{1-\rho\sigma_e\sigma_c\gamma^{-1}\nu}\Theta(\overline{R}_0)=\frac{1}{1-\rho\sigma_e\sigma_c\gamma^{-1}\nu}\Theta(\overline{R}_0)+\left[\overline{R}_0\delta(\overline{R}_0)\text{-term}\right]$$

$$\implies\left\langle\frac{\partial\overline{R}_0}{\partial K}\right\rangle=\boxed{\chi=\frac{\phi_R}{1-\rho\sigma_e\sigma_c\gamma^{-1}\nu}} \tag{A42}$$

We can solve these two equations for χ,ν to obtain the relations,

$$\nu=\frac{\gamma^{-1}\phi_N/\phi_R}{\rho\sigma_c\sigma_e\gamma^{-1}(\gamma^{-1}\phi_N/\phi_R-1)},\qquad\chi=\phi_R-\gamma^{-1}\phi_N. \tag{A43}$$

Next, we use Eqs. A20 and A28 and invoke our assumption that the system self-averages to find,

$$\boxed{\langle N\rangle=\langle\overline{N}_0\rangle=\frac{\sigma_g}{\sigma_c\sigma_e\rho\chi}W_1(\Delta_g,1)=\frac{\sigma_g}{\sigma_c\sigma_e\rho\chi}\left(\frac{e^{-\Delta_g^2/2}}{\sqrt{2\pi}}+\Delta_g\Phi(\Delta_g)\right)}, \tag{A44}$$

$$\boxed{\langle R\rangle=\langle\overline{R}_0\rangle=\frac{\sigma_\kappa}{1-\rho\sigma_e\sigma_c\gamma^{-1}\nu}W_1(\Delta_\kappa,1)=\frac{\sigma_\kappa}{1-\rho\sigma_e\sigma_c\gamma^{-1}\nu}\left(\frac{e^{-\Delta_\kappa^2/2}}{\sqrt{2\pi}}+\Delta_\kappa\Phi(\Delta_\kappa)\right)}, \tag{A45}$$

$$\boxed{q_N=\langle\overline{N}_0^2\rangle=\left(\frac{\sigma_g}{\sigma_c\sigma_e\rho\chi}\right)^2W_2(\Delta_g,1)=\left(\frac{\sigma_g}{\sigma_c\sigma_e\rho\chi}\right)^2\left(\frac{\Delta_g e^{-\Delta_g^2/2}}{\sqrt{2\pi}}+(1+\Delta_g^2)\Phi(\Delta_g)\right)}, \tag{A46}$$

$$\boxed{q_R=\langle\overline{R}_0^2\rangle=\left(\frac{\sigma_\kappa}{1-\rho\sigma_e\sigma_c\gamma^{-1}\nu}\right)^2W_2(\Delta_\kappa,1)=\left(\frac{\sigma_\kappa}{1-\rho\sigma_e\sigma_c\gamma^{-1}\nu}\right)^2\left(\frac{\Delta_\kappa e^{-\Delta_\kappa^2/2}}{\sqrt{2\pi}}+(1+\Delta_\kappa^2)\Phi(\Delta_\kappa)\right)}. \tag{A47}$$

The equations A39, A40, A41, A42, A44, A45, A46, A47 constitute the cavity self-consistency equations for the aMCRM model. They are eight independent nonlinear equations to solve for eight variables: $\phi_N,\phi_R,\chi,\nu,\langle N\rangle,\langle R\rangle,q_N,q_R$. These equations are solved numerically using nonlinear least squares as discussed in SI section D 3.

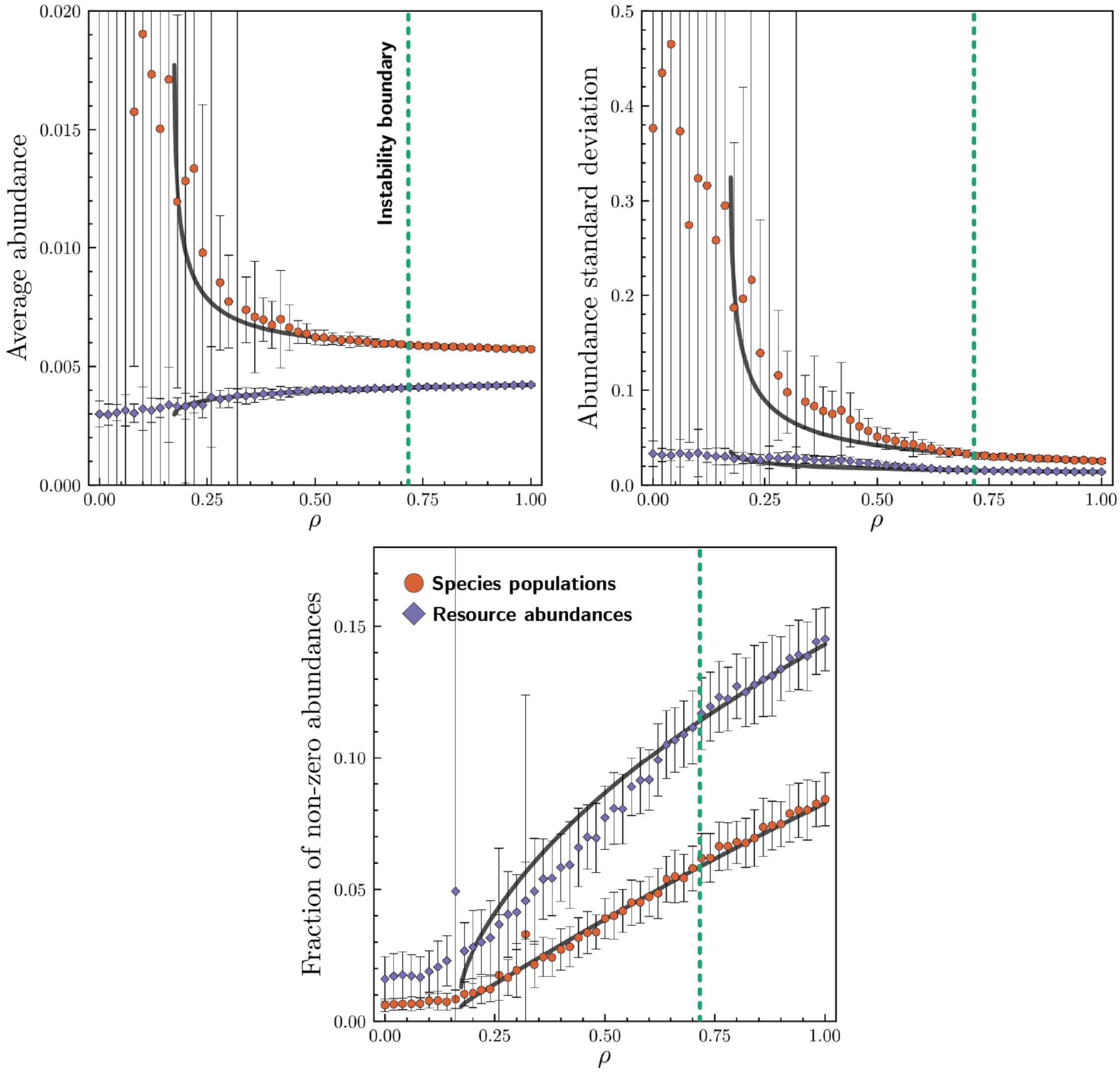

FIG. A6. Comparison of cavity results to numerical simulations for various ρ and fixed $\sigma_c = \sigma_e = 3.5$, corresponding to the gray dashed line in Fig. 3. (a) Average populations of species, $\langle N \rangle$, and abundances of resources, $\langle R \rangle$. (b) Standard deviations of species populations, $\sqrt{\langle N^2 \rangle - \langle N \rangle^2}$, and resource abundances, $\sqrt{\langle R^2 \rangle - \langle R \rangle^2}$. (c) Fractions of surviving species, ϕ_N, and non-depleted resources, ϕ_R. Points are means of numerical simulations, lines are cavity results. Error bars are standard deviations of numerical simulations; some error bars are smaller than the points. A dashed line is shown at $\rho^\star = 0.72$, the critical value of ρ at which the system transitions between the stable and dynamic phases. For each value of ρ, 64 simulations are performed with $M, S = 512$ resources and species.

5. Infeasibility of the cavity solution

Next, we will investigate when there may exist a solution to the cavity self-consistency equations. Observe that Eq. (A41) implies that as $\chi \to 0$, ν diverges; Eqs. A21, A29 indicate that $\overline{N}_0$ and $\overline{R}_0$ become singular. This is when, numerically, there fails to exist a solution to the cavity self-consistency equations. From Eq. (A42), we see that $\chi \to 0$ when,

$$\phi_R^{\text{CSI}} - \gamma^{-1}\phi_N^{\text{CSI}} = 0 \Longleftrightarrow \frac{\phi_R^{\text{CSI}}}{\phi_N^{\text{CSI}}}\frac{M}{S} = 1 \tag{A48}$$

$$\Downarrow$$

$$\boxed{\text{cavity solution infeasibility boundary:} \quad \#\text{ of non-depleted resources} = \#\text{ of surviving species.}} \tag{A49}$$

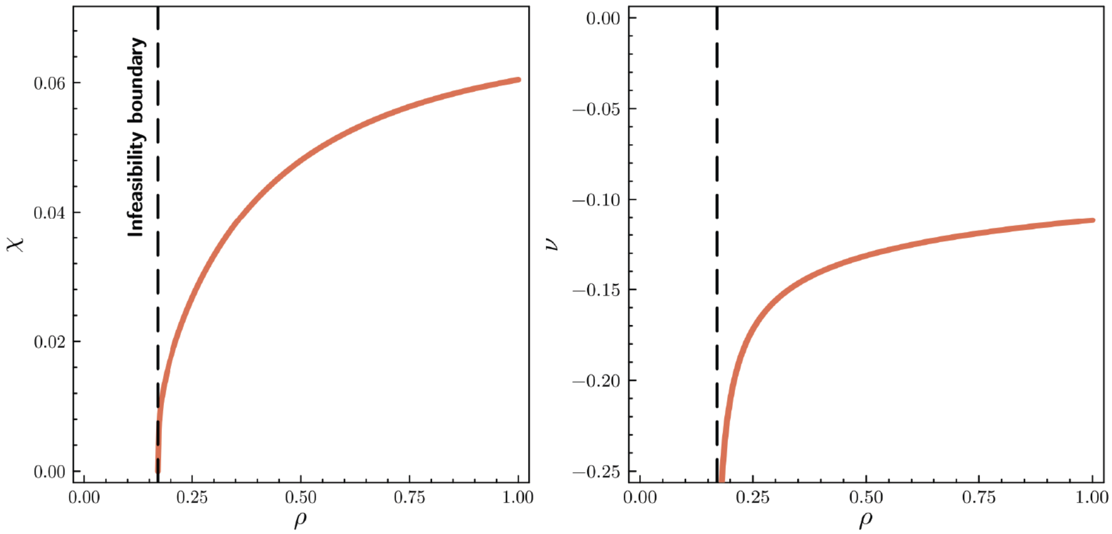

FIG. A7. Cavity susceptibilities for the same parameters as in Fig. A6. The susceptibilities χ, ν as defined in lines A16, A24 are shown on the left and right, respectively.

The cavity solution no longer has a solution when the ecosystem approaches the bound set by the principle of competitive exclusion. According to this calculation, ν diverging here means that if a species becomes marginally more fit when all niches are packed, it will disrupt the ecosystem. This relation defines a ninth equation for eight variables, so it determines a co-dimension-one boundary in the space of parameters. This boundary can be calculated numerically using nonlinear least squares as discussed in SI section D 3. When solving the cavity self-consistency equations numerically, we will see that there is a region of parameter space beyond this boundary where the least squares objectives are large compared to machine error (see Fig. D15 in SI section D 3).

This boundary corresponds to the transition to the "unbounded growth" phase in the Lotka–Volterra literature (see Ref. [31]) because $\langle N\rangle \to \infty$ as $\chi \to 0$ as can be seen in Eq. (A44). There is no unbounded growth in this model due to the negative feedback structure of consumer-resource models. In the cavity solution, this can be seen through $\langle R\rangle \to 0$ as $\nu \to -\infty$ in Eq. (A45). That is, species abundances are predicted to diverge while abundances of all resources become zero. Additionally, as $\chi \to 0$, $q_R \to 0$, so $\sigma_g = \sigma_m$ and $g = -m$, meaning $\phi_N^{\mathrm{CSI}} = 1 - \Phi(m/\sigma_m)$; therefore ϕ_N and ϕ_R approach nonzero values at this boundary.

6. Comparison of cavity and simulation results

For higher reciprocity levels ρ when the system is in the stable phase, the analytic solution produced from the cavity method matches the simulation results remarkably well. In Fig. A6, we compare the cavity results to numerical simulations for various $0 \le \rho \le 1$ and fixed $\sigma_c = \sigma_e = 3.5$, corresponding to the gray dashed line in Fig. 3(a). For all predicted quantities, the chi-squared statistic is near the number of degrees of freedom, indicating that the cavity solution is a good fit to the simulation results. The cavity susceptibilities χ, ν for this slice are shown in Fig. A7. In this figure, we see that the infeasibility boundary occurs when $\chi \to 0$ and $\nu \to -\infty$.

In Fig. A8, we compare the cavity results to numerical simulations for the various values ρ and $\sigma_c = \sigma_e \equiv \sigma$ shown on the grid in Fig. 3(a). Again, the analytic solutions produced from the cavity method match the simulation results remarkably well in the stable phase. Cavity susceptibilities χ, ν for this grid are shown in Fig. A9.

7. Cavity susceptibilities

The cavity susceptibilities,

$$\chi \equiv \frac{1}{M}\sum_{\alpha=1}^{M}\frac{\partial R_\alpha}{\partial K_\alpha}, \qquad \nu \equiv \frac{1}{S}\sum_{i=1}^{S}\frac{\partial N_i}{\partial m_i}, \tag{A50}$$

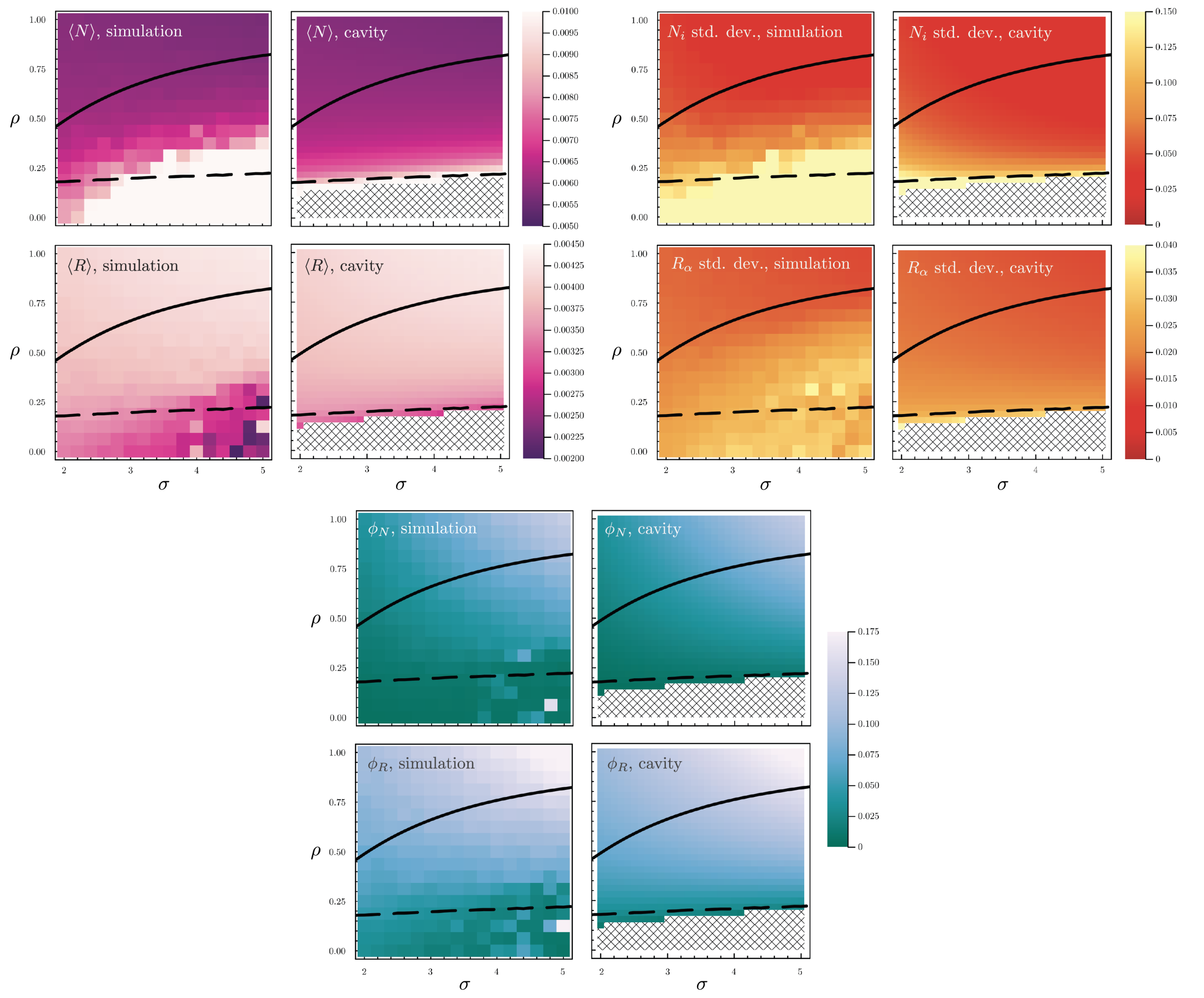

FIG. A8. Heatmap comparison of cavity predictions and simulation results. Results from 32 simulations for various values of σ and ρ are displayed in the left column of each pane and the cavity predictions are displayed in the right column. The instability boundary is shown as a solid black line while the infeasibility boundary is shown as a dashed black line. For the cavity prediction plots, the parameter regions for which no cavity solution exists have a diamond pattern overlaid (c.f., Fig. D15 and SI section D 3). The parameters used are those used in Fig. 3(a).

have physical significance: χ and ν are the average linear-order response of a resource's abundance to a small change in its carrying capacity and a species' population to a small change in its natural mortality rate, respectively. These susceptibilities could be measured numerically by introducing small, nonzero-mean perturbations to K_α and measuring $\sum_{\alpha=1}^{M} \Delta R_\alpha / \sum_{\alpha=1}^{M} \Delta K_\alpha$ when the system is self-averaging.

At steady state, the full susceptibility matrices can be computed using just the knowledge of which species and resources. Consider that the surviving species and resources satisfy the following matrix equation:

$$\begin{bmatrix} 0 & c^* \\ (e^*)^{\mathrm{T}} & 1 \end{bmatrix} \begin{bmatrix} N^* \\ R^* \end{bmatrix} = \begin{bmatrix} m^* \\ K^* \end{bmatrix}, \tag{A51}$$

where X^* represents a matrix or vector with rows and/or columns corresponding to species and resource which are not surviving removed. This block-matrix equation can be solved to find,

$$\begin{bmatrix} N^* \\ R^* \end{bmatrix} = \begin{bmatrix} -\left(c^*(e^*)^{\mathrm{T}}\right)^{-1} & \left(c^*(e^*)^{\mathrm{T}}\right)^{-1} c^* \\ (e^*)^{\mathrm{T}}\left(c^*(e^*)^{\mathrm{T}}\right)^{-1} & 1 - (e^*)^{\mathrm{T}}(c^*(e^*)^{\mathrm{T}})^{-1} c^* \end{bmatrix} \begin{bmatrix} m^* \\ K^* \end{bmatrix}. \tag{A52}$$

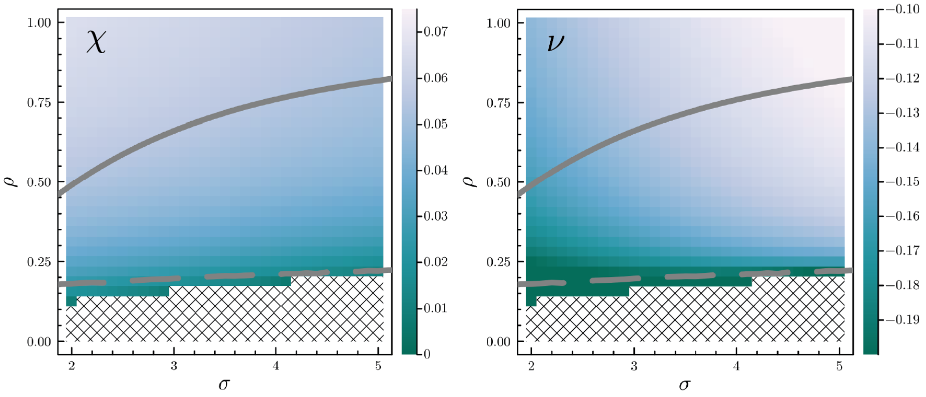

FIG. A9. Heatmap of cavity susceptibilities χ, ν as defined in lines A16, A24 for various values of σ and ρ, as in Fig. A8.

Given that perturbations are sufficiently small so that no species or resources go extinct and no new species or resources can invade, the susceptibility matrices are,

$$\begin{aligned} [\nu_{ij}^{(N)}] &= -\big(c^*(e^*)^{\mathrm{T}}\big)^{-1}, & [\chi_{i\beta}^{(N)}] &= \big(c^*(e^*)^{\mathrm{T}}\big)^{-1}c^*, \\ [\nu_{\alpha j}^{(R)}] &= (e^*)^{\mathrm{T}}\big(c^*(e^*)^{\mathrm{T}}\big)^{-1}, & [\chi_{\alpha\beta}^{(R)}] &= 1-(e^*)^{\mathrm{T}}(c^*(e^*)^{\mathrm{T}})^{-1}c^*. \end{aligned} \tag{A53}$$

Therefore,

$$\nu = -\frac{1}{S}\,\mathrm{tr}\,\big(c^*(e^*)^{\mathrm{T}}\big)^{-1}, \qquad \chi = \frac{1}{M}\,\mathrm{tr}\left[1-(e^*)^{\mathrm{T}}(c^*(e^*)^{\mathrm{T}})^{-1}c^*\right]. \tag{A54}$$

This means that when a steady state exists, the cavity susceptibilities can be computed exactly with knowledge of which species and resources are surviving. Notice that $(e^*)^{\mathrm{T}}(c^*(e^*)^{\mathrm{T}})^{-1}c^*$ is an oblique projector because it is idempotent. The trace of this matrix is the dimension of the range of the projector. Therefore, the infeasibility boundary in the cavity solution occurs (or equivalently, the niches are fully packed) when the projector is full-rank.

Appendix B: Stability phase transition in the thermodynamic limit

In nonlinear dynamics, a common approach to assess the appearance of instability is to begin with an assumption of stability and see what breaks down when the system is perturbed. In the case of the aMCRM, when there is stability, the results from the cavity solution will be valid, so we will use these results to determine the critical threshold for instability.

We consider perturbing all surviving species and resources, $\overline{N}_i^+$, $\overline{R}_\alpha^+$, by small amounts, $\varepsilon\eta_i^{(N)}$, $\varepsilon\eta_\alpha^{(R)}$, respectively, where $\eta_i^{(N)}$ and $\eta_\alpha^{(R)}$ are independent random variables all with mean zero and variance one [31]. By considering perturbations on surviving species and resources and using cavity results (c.f., Eqs. A29, A21), we are assuming that we are working with an *uninvadable* steady state. From Eqs. A17, A25, for surviving species and non-depleted resources,

$$\overline{N}_0^+ = \frac{1}{\sigma_c\sigma_e\rho\chi}\left[g+\frac{\sigma_c}{\sqrt{M}}\sum_{\alpha:\overline{R}_\alpha>0} d_{0\alpha}\overline{R}_{\alpha\backslash 0}^+ - \sigma_m\delta m_0\right], \tag{B1}$$

$$\overline{R}_0^+ = \frac{1}{1-\rho\sigma_e\sigma_c\gamma^{-1}\nu}\left[\kappa-\frac{\sigma_e}{\sqrt{M}}\sum_{i:\overline{N}_i>0}\overline{N}_{i\backslash 0}^+\left(\rho d_{i0}+\sqrt{1-\rho^2}x_{i0}\right)+\sigma_K\delta K_0\right]. \tag{B2}$$

Applying the perturbation gives,

$$\overline{N}_0^+ = \frac{1}{\sigma_c\sigma_e\rho\chi}\left[g + \frac{\sigma_c}{\sqrt{M}}\sum_{\alpha:\overline{R}_\alpha>0} d_{0\alpha}\left(\overline{R}_{\alpha\backslash 0}^+ + \varepsilon\eta_\alpha^{(R)}\right) - \sigma_m\delta m_0\right], \tag{B3}$$

$$\overline{R}_0^+ = \frac{1}{1-\rho\sigma_e\sigma_c\gamma^{-1}\nu}\left[\kappa - \frac{\sigma_e}{\sqrt{M}}\sum_{i:\overline{N}_i>0}\left(\overline{N}_{i\backslash 0}^+ + \varepsilon\eta_i^{(N)}\right)\left(\rho d_{i0} + \sqrt{1-\rho^2}x_{i0}\right) + \sigma_K\delta K_0\right]. \tag{B4}$$

Differentiating with respect to ε yields,

$$\frac{\mathrm{d}\overline{N}_0^+}{\mathrm{d}\varepsilon} = \frac{1}{\sigma_e\rho\chi\sqrt{M}}\sum_{\alpha:\overline{R}_\alpha>0} d_{0\alpha}\left(\frac{\mathrm{d}\overline{R}_{\alpha\backslash 0}^+}{\mathrm{d}\varepsilon} + \eta_\alpha^{(R)}\right), \tag{B5}$$

$$\frac{\mathrm{d}\overline{R}_0^+}{\mathrm{d}\varepsilon} = -\frac{\sigma_e/\sqrt{M}}{1-\rho\sigma_e\sigma_c\gamma^{-1}\nu}\sum_{i:\overline{N}_i>0}\left(\frac{\mathrm{d}\overline{N}_{i\backslash 0}^+}{\mathrm{d}\varepsilon} + \eta_i^{(N)}\right)\left(\rho d_{i0} + \sqrt{1-\rho^2}x_{i0}\right). \tag{B6}$$

Because $\overline{R}_{\alpha\backslash 0}^+$, $d_{0\alpha}$, $\eta_\alpha^{(R)}$ are all independent, $\langle d\overline{R}_0^+/\mathrm{d}\varepsilon\rangle = 0$; similarly, $\langle d\overline{N}_0^+/\mathrm{d}\varepsilon\rangle = 0$. The first moment of the response to the perturbation does not give useful information about the stability; therefore, we turn to the second moment. First, we square the above quantities:

$$\left[\frac{\mathrm{d}\overline{N}_0^+}{\mathrm{d}\varepsilon}\right]^2 = \frac{1/M}{(\sigma_e\rho\chi)^2}\sum_{\alpha,\beta:\overline{R}_\alpha>0,\overline{R}_\beta>0} d_{0\alpha}d_{0\beta}\left(\frac{\mathrm{d}\overline{R}_{\alpha\backslash 0}^+}{\mathrm{d}\varepsilon} + \eta_\alpha^{(R)}\right)\left(\frac{\mathrm{d}\overline{R}_{\beta\backslash 0}^+}{\mathrm{d}\varepsilon} + \eta_\beta^{(R)}\right), \tag{B7}$$

$$\left[\frac{\mathrm{d}\overline{R}_0^+}{\mathrm{d}\varepsilon}\right]^2 = \frac{\sigma_e^2/M}{(1-\rho\sigma_e\sigma_c\gamma^{-1}\nu)^2}\sum_{i,j:\overline{N}_i>0,\overline{N}_j>0}\left(\frac{\mathrm{d}\overline{N}_{i\backslash 0}^+}{\mathrm{d}\varepsilon} + \eta_i^{(N)}\right)\left(\frac{\mathrm{d}\overline{N}_{j\backslash 0}^+}{\mathrm{d}\varepsilon} + \eta_j^{(N)}\right) \tag{B8}$$
$$\times\left(\rho d_{i0} + \sqrt{1-\rho^2}x_{i0}\right)\left(\rho d_{j0} + \sqrt{1-\rho^2}x_{j0}\right).$$

Averaging over all sources of randomness and using $\left\langle\left[\frac{\mathrm{d}N_0}{\mathrm{d}\varepsilon}\right]^2\right\rangle = S^{-1}\sum_{i=1}^S\left[\frac{\mathrm{d}N_{i\backslash 0}}{\mathrm{d}\varepsilon}\right]^2$ and $\left\langle\left[\frac{\mathrm{d}R_0}{\mathrm{d}\varepsilon}\right]^2\right\rangle = M^{-1}\sum_{\alpha=1}^M\left[\frac{\mathrm{d}R_{\alpha\backslash 0}}{\mathrm{d}\varepsilon}\right]^2$, which follows from the assumption that the system self averages, we obtain:

$$\left\langle\left[\frac{\mathrm{d}\overline{N}_0^+}{\mathrm{d}\varepsilon}\right]^2\right\rangle = \frac{1/M}{(\sigma_e\rho\chi)^2}\sum_{\alpha,\beta:\overline{R}_\alpha>0,\overline{R}_\beta>0}\langle d_{0\alpha}d_{0\beta}\rangle\left(\frac{\mathrm{d}\overline{R}_{\alpha\backslash 0}^+}{\mathrm{d}\varepsilon}\frac{\mathrm{d}\overline{R}_{\beta\backslash 0}^+}{\mathrm{d}\varepsilon} + \frac{\mathrm{d}\overline{R}_{\alpha\backslash 0}^+}{\mathrm{d}\varepsilon}\left\langle\eta_\beta^{(R)}\right\rangle\right. \tag{B9}$$
$$\left. + \left\langle\eta_\alpha^{(R)}\right\rangle\frac{\mathrm{d}\overline{R}_{\beta\backslash 0}^+}{\mathrm{d}\varepsilon} + \left\langle\eta_\alpha^{(R)}\eta_\beta^{(R)}\right\rangle\right)$$

$$= \frac{1/M}{(\sigma_e\rho\chi)^2}\sum_{\alpha,\beta:\overline{R}_\alpha,\overline{R}_\beta>0}\delta_{\alpha\beta}\left(\frac{\mathrm{d}\overline{R}_\alpha\backslash}{\mathrm{d}\varepsilon}\frac{\mathrm{d}\overline{R}_{\beta\backslash 0}}{\mathrm{d}\varepsilon} + \delta_{\alpha\beta}\right) \tag{B10}$$

$$= \frac{\phi_R}{(\sigma_e\rho\chi)^2}\left(\left\langle\left[\frac{\mathrm{d}\overline{R}_0^+}{\mathrm{d}\varepsilon}\right]^2\right\rangle + 1\right), \tag{B11}$$

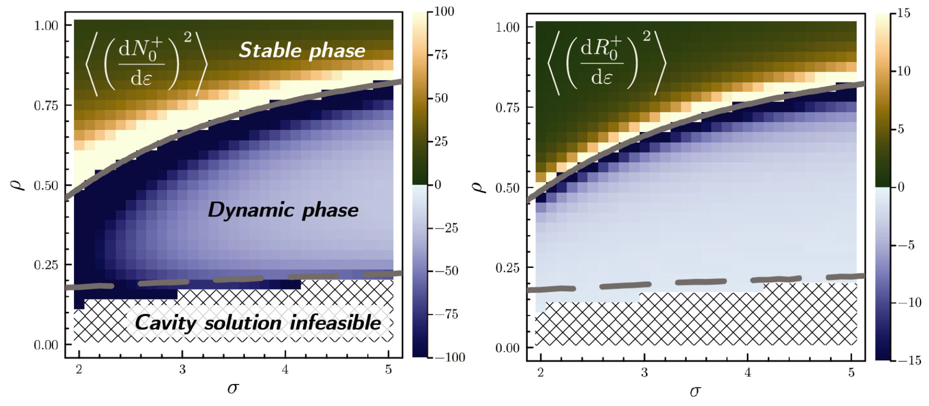

FIG. B10. Heatmaps of the variances of the response of a surviving species and resources to a random perturbation (Eqs. B15, B16) as a function of the parameters σ and ρ with other parameters matching those in Fig. 3(a). The instability boundary is shown as a gray solid line, and the infeasibility boundary is shown as a gray dashed line.

$$\left\langle \left[\frac{\mathrm{d}\overline{R}_0^+}{\mathrm{d}\varepsilon}\right]^2 \right\rangle = \frac{\sigma_e^2/M}{(1-\rho\sigma_e\sigma_c\gamma^{-1}\nu)^2} \sum_{i,j:\overline{N}_i>0,\overline{N}_j>0} \left(\frac{\mathrm{d}\overline{N}_{i\backslash 0}^+}{\mathrm{d}\varepsilon}\frac{\mathrm{d}\overline{N}_{j\backslash 0}^+}{\mathrm{d}\varepsilon} + \frac{\mathrm{d}\overline{N}_{i\backslash 0}^+}{\mathrm{d}\varepsilon}\left\langle \eta_j^{(N)}\right\rangle + \left\langle \eta_i^{(N)}\right\rangle \frac{\mathrm{d}\overline{N}_{j\backslash 0}^+}{\mathrm{d}\varepsilon} + \left\langle \eta_i^{(N)}\eta_j^{(N)}\right\rangle \right) \times \left(\rho^2 \langle d_{i0}d_{j0}\rangle + \rho\sqrt{1-\rho^2}\left(\langle d_{i0}\rangle\langle x_{j0}\rangle + \langle x_{i0}\rangle\langle d_{j0}\rangle\right) + (1-\rho^2)x_{i0}x_{j0}\right) \tag{B12}$$

$$= \frac{\sigma_e^2(S/M)/S}{(1-\rho\sigma_e\sigma_c\gamma^{-1}\nu)^2} \sum_{i,j:\overline{N}_i>0,\overline{N}_j>0} \left(\frac{\mathrm{d}\overline{N}_{i\backslash 0}^+}{\mathrm{d}\varepsilon}\frac{\mathrm{d}\overline{N}_{j\backslash 0}^+}{\mathrm{d}\varepsilon} + \delta_{ij}\right)\left(\rho^2\delta_{ij} + (1-\rho^2)\delta_{ij}\right) \tag{B13}$$

$$= \frac{\sigma_e^2\gamma^{-1}\phi_N}{(1-\rho\sigma_e\sigma_c\gamma^{-1}\nu)^2} \left(\left\langle \left[\frac{\mathrm{d}\overline{N}_0^+}{\mathrm{d}\varepsilon}\right]^2 \right\rangle + 1 \right). \tag{B14}$$

Solving this system of equations yields:

$$\boxed{\left\langle \left[\frac{\mathrm{d}\overline{N}_0^+}{\mathrm{d}\varepsilon}\right]^2 \right\rangle = \frac{\phi_R\left((1-\nu\rho\sigma_c\sigma_e\gamma^{-1})^2\sigma_e^{-2} + \gamma^{-1}\phi_N\right)}{\left[\rho\chi\left(1-\nu\rho\sigma_c\sigma_e\gamma^{-1}\right)\right]^2 - \gamma^{-1}\phi_N\phi_R}}, \tag{B15}$$

$$\boxed{\left\langle \left[\frac{\mathrm{d}\overline{R}_0^+}{\mathrm{d}\varepsilon}\right]^2 \right\rangle = \frac{\gamma^{-1}\phi_N\left(\phi_R + (\rho\sigma_e\chi)^2\right)}{\left[\rho\chi\left(1-\nu\rho\sigma_c\sigma_e\gamma^{-1}\right)\right]^2 - \gamma^{-1}\phi_N\phi_R}}. \tag{B16}$$

These susceptibilities are the variance of the response of a surviving species or resource when the system is subject to a random perturbation. The divergence of these variances represent the breakdown of the mean-field approximation. Further, when these susceptibilities diverge, the uninvadable fixed point predicted by the mean-field approximation becomes dynamically unstable. Therefore, when these susceptibilities are divergent, whenever invasion is attempted, the system will exhibit dynamical fluctuations. We call the boundary in parameter space at which these susceptibilities diverge the instability boundary. These susceptibilities diverge when $0 = \left[\rho\chi\left(1-\nu\rho\sigma_c\sigma_e\gamma^{-1}\right)\right]^2 - \gamma^{-1}\phi_N\phi_R$. This condition along with the cavity self-consistency equations (Eqs. A39, A40, A41, A42, A44, A45, A46, A47) determines a co-dimension-one boundary in the space of model parameters. By using the relations in line A43, we obtain the

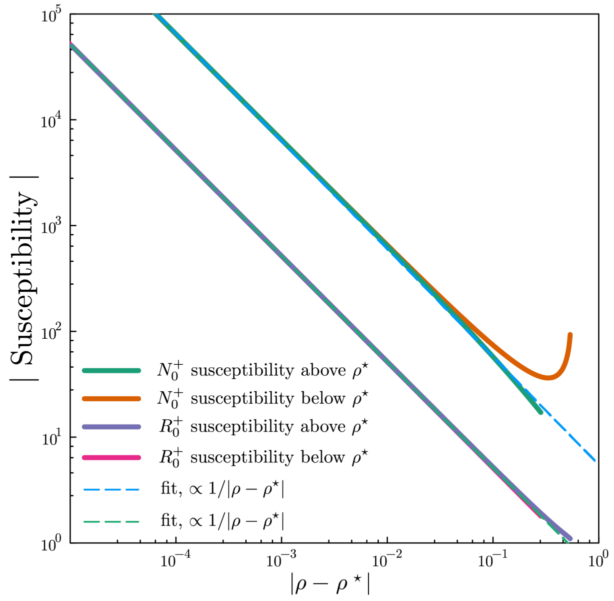

FIG. B11. Log-log plot of the absolute value of the variances of the susceptibilities $\langle (dN_0^+/\mathrm{d}\varepsilon)^2 \rangle$, $\langle (dR_0^+/\mathrm{d}\varepsilon)^2 \rangle$ plotted as a function of the distance from the instability boundary, $|\rho - \rho^\star|$. The data is exactly that which is used in Fig. 3(b). A fit $\propto 1/|\rho - \rho^\star|$ is shown for each susceptibility with dashed lines.

following relation for the boundary at which the system becomes unstable:

$$\phi_R^\star \left((\rho^\star)^2 \phi_R^\star - \gamma^{-1} \phi_N^\star \right) = 0 \implies (\rho^\star)^2 = \gamma^{-1} \frac{\phi_N^\star}{\phi_R^\star} \tag{B17}$$

$$\Downarrow$$

$$\boxed{\text{instability boundary occurs when:} \quad (\rho^\star)^2 = \frac{(\#\text{ of surviving species})^\star}{(\#\text{ of non-depleted resources})^\star},} \tag{B18}$$

where $X^\star$ denotes the value of a quantity X at the instability boundary. We are able to determine the critical level of asymmetry, $\rho^\star$, at which the ecosystem becomes unstable by solving a nonlinear least squares problem (see SI section D 3). The variances of the susceptibilities $\langle (\mathrm{d}N_0^+/\mathrm{d}\varepsilon)^2 \rangle$, $\langle (\mathrm{d}R_0^+/\mathrm{d}\varepsilon)^2 \rangle$ are shown for various values of $\sigma = \sigma_c = \sigma_e$ and ρ in Fig. B10 with the instability and infeasibility boundaries overlaid. These variances of susceptibilities additionally have an asymptotic power law dependence on the distance from the instability boundary, $|\rho - \rho^\star|$, as shown in Fig. B11.

The transition to the dynamic phase coincides in method to the transition to the "multiple attractors" phase discussed in the random generalized Lotka–Volterra model literature. This was first described in Ref. [31].

Appendix C: Chaotic nature of dynamics

In the dynamic phase of the aMCRM, when an ecosystem is sufficiently large, the dynamics are chaotic. In this section, we provide numerical evidence that the dynamics of the aMCRM are chaotic in the classical sense (i.e., sensitive dependence on initial conditions) and in the sense of unpredictability (i.e., no clear patterns in the dynamics). As shown in Fig. 4(b) in the main text, the trajectories of the abundances of the surviving species and resources diverge from one another, indicating that the dynamics are chaotic in the classical sense. Evidence of chaos and unpredictability in the dynamic phase is present in other numerical experiments, as well.

In Fig. C12, we show that the dynamics of the aMCRM in the dynamic phase are unpredictable in the sense that a trajectory does not display any clear patterns in the projection onto the first three principal components of the correlation matrix of the time series of the abundances of the surviving species and resources. Such a projection is necessary to visualize the dynamics of the system, as they are very high-dimensional.

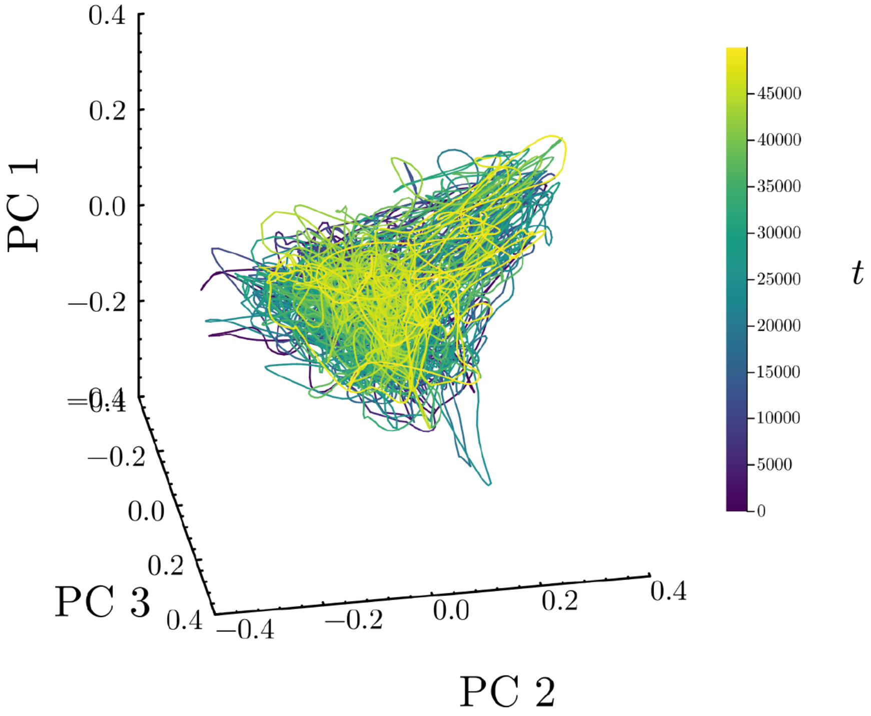

FIG. C12. Projection of $M + S = 256 + 256 = 512$-dimensional dynamics onto the 3 highest-ranked eigenvectors of the correlation matrix (first 3 principal components) of the time series of the abundances. The lack of clustering of the trajectories in the projection indicates that the dynamics are chaotic and unpredictable. The parameters correspond to the gray star in Fig. 3(a). Before plotting, the simulation was run for 10^4 time units to eliminate any potential transients.

1. Analysis of the Lyapunov exponents

In Fig. 4(a), we show the maximal Lyapunov exponent for simulations classified by whether they reach a steady state for various values of ρ. To determine the Lyapunov spectrum, we use the `lyapunovspectrum` function in the `ChaosTools.jl` *Julia* package which employs the 'H2' method of Geist, originally stated in Benettin et al. [39–42]. This algorithm is described in detail in SI section A of Ref. [41], and its applications are described in Chapter 3.2. Conceptually, in order to compute the kth largest Lyapunov exponent, the algorithm evolves $n \geq k$ deviation vectors in the tangent space of the system and computes how the shape of the n-dimensional parallelepiped spanned by the deviation vectors evolve; the eigenvalues of the matrix describing the evolution of the parallelepiped are asymptotically related to the Lyapunov exponents. The Jacobian of the system when the dynamical variable is considered as $(N_1, \ldots, N_S, R_1, \ldots, R_M)$ is:

$$\mathbf{J}(N_1, \ldots, N_S, R_1, \ldots, R_M) = \left[\begin{array}{c|c} \frac{\partial \dot{N}_i}{\partial N_j} & \frac{\partial \dot{N}_i}{\partial R_\beta} \\ \hline \frac{\partial \dot{R}_\alpha}{\partial N_j} & \frac{\partial \dot{R}_\alpha}{\partial R_\beta} \end{array}\right] = \left[\begin{array}{c|c} \delta_{ij}(\sum_{i=1}^S c_{i\alpha} R_\alpha - m_i) & N_i c_{i\beta} \\ \hline -e_{j\alpha} R_\alpha & \delta_{\alpha\beta}(K_\alpha - 2R_\alpha - \sum_{i=1}^S c_{i\alpha} N_i) \end{array}\right]. \quad \text{(C1)}$$

The Lyapunov spectrum is computed with $M = S = 512$ species and resources, and the initial deviation vectors are chosen to be 8 unit vectors in the direction of randomly chosen species and resources. The parameters passed to the `lyapunovspectrum` function are the number of steps, `N = 1000`, the time step, `Δt = 5`, and the time to wait before starting to record the Lyapunov spectrum, `Ttr = 1000`. The initial conditions are $N_i(0) = 1/S$ and $R_\alpha(0) = 1/M$.

As seen in Fig. 4(a), the maximal Lyapunov exponent for a simulation that does not reach a steady state is positive while the maximal Lyapunov exponent for a simulation that does reach a steady state is negative. A simulation is categorized as reaching a steady state if the mean absolute value of all derivatives of the abundances at the end of the simulation is less than 10^{-6}. These results indicates that the dynamics are chaotic in the classical sense for the simulations which have persistent fluctuations. The impact of system size on the probability of reaching a steady state is discussed in SI section E.

2. Analysis of the generalized alignment index (GALI)

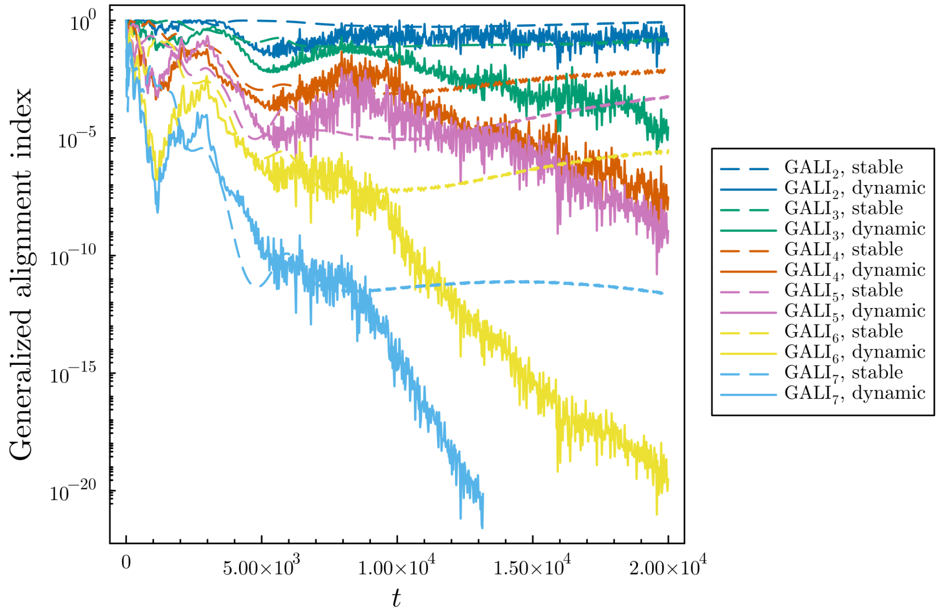

FIG. C13. Generalized alignment indices (GALIs) for simulations in the dynamic and stable phases with $M = S = 512$ species and resources. The simulations all have the same sampled parameters; only ρ and order of the GALI are varied. For the simulations in the dynamic phase (solid lines), the GALI asymptotically decays exponentially with time, indicating chaos, while for the simulations in the stable phase (dashed lines), the GALI asymptotically approaches a nonzero value, indicating the dynamics achieve a steady state. Simulation parameters for the GALI in the stable and dynamic phases correspond to the gray dot and star in Fig. 3(a), respectively.

An alternative method to determine whether a system is chaotic is to use the generalized alignment index (GALI) [41–43]. The GALI is a measure of how vectors in the tangent space of a trajectory align with each other. Let $\hat{w}_1(0), \ldots, \hat{w}_k(0) \in \mathbb{R}^{M+S}$ be linearly-independent unit deviation vectors that evolve according to,

$$\frac{\mathrm{d}}{\mathrm{d}t}\hat{w}_i(t) = \mathbf{J}(x(t))\hat{w}_i(t), \tag{C2}$$

where $x(t) = (N_1(t), \ldots, N_S(t), R_1(t), \ldots, R_M(t))$ and $\mathbf{J}(x(t))$ is the Jacobian matrix of the system in state $x(t)$. These deviation vectors are normalized to have unit length at regular (small) time intervals. The order-k GALI is defined as,

$$\text{GALI}_k(t) = \|\hat{w}_1(t) \wedge \hat{w}_2(t) \wedge \cdots \wedge \hat{w}_k(t)\| = |\det(\hat{w}_1(t), \hat{w}_2(t), \ldots, \hat{w}_k(t))|, \tag{C3}$$

which is the volume of the k-dimensional parallelepiped spanned by the time-evolved deviation vectors. If the system is chaotic, the GALI will decay exponentially with time, indicating that the deviation vectors are becoming more aligned with each other due to the exponential divergence of nearby trajectories. If the system is not chaotic and reaches a steady state, the GALI will asymptotically approach a nonzero value, indicating that the deviation vectors form a nonzero volume in the tangent space because no single direction dominates the dynamics. Alternatively, if the system is asymptotically periodic or quasi-periodic and does not reach a steady state, the GALI will decay as a power law with time.

In Fig. C13, we show the GALI for simulations in the dynamic and stable phases for $k = 2, \ldots, 7$ with $M = S = 512$ species and resources. We use the `gali` function from the `ChaosTools.jl` *Julia* package [42]. The simulations all have the same sampled parameters; only ρ and the order of the GALI are varied. For the simulations in the dynamic phase (solid lines), the GALI asymptotically decays exponentially with time, indicating chaos, while for the simulations in the stable phase (dashed lines), the GALI asymptotically approaches a nonzero value, indicating the dynamics achieve a steady state. The initial deviation vectors are chosen to be k unit vectors in the direction of randomly chosen species and resources. The arguments passed to the `gali` function are the run time, `T = 2e4`, the time step, `Δt = 5.`, the threshold at which to stop the simulation, `threshold = 1e-22`, and the order of the GALI, `k`.

3. Analysis of effective dimension of dynamics

Chaotic systems can often be characterized by their effective dimension, which is the number of degrees of freedom that are relevant to the dynamics. One measure of the effective dimension is the Kaplan–Yorke (KY) dimension [44, 57, 58], which is the number of Lyapunov exponents that are positive. The KY dimension is the linearly interpolated point at which the cumulative sum of the Lyapunov exponents crosses zero:

$$D_{\mathrm{KY}} = k + \frac{\sum_{i=1}^{k} \lambda_i}{|\lambda_{k+1}|}, \qquad k = \max_j \left(\sum_{i=1}^{j} \lambda_i > 0 \right) \tag{C4}$$

We estimate the KY dimension of the aMCRM using the Lyapunov spectrum measured using the methods described in SI section C 1 and the `kaplanyorke_dim` function from the `FractalDimensions.jl` *Julia* package [58].

We find that the KY dimension of the aMCRM in the dynamic phase for the parameters corresponding to the gray star in Fig. 3(a) is $D_{\mathrm{KY}} = 15 \pm 7$, which is an average over 64 simulations. The number of surviving species and resources in these simulations at the end of the simulation is $S^\star + M^\star = 74 \pm 17$. The ratio of the KY dimension to the number of surviving species and resources is $D_{\mathrm{KY}}/(S^\star + M^\star) = 0.21 \pm 0.09$. The number of species and resources that are transitioning between high- and low-abundance states is $M_t + S_t = 38 \pm 19$, and the ratio of the KY dimension to the number of species and resources that are transitioning between high- and low-abundance states is $D_{\mathrm{KY}}/(M_t + S_t) = 2.6 \pm 1.2 \approx O(1)$. From this, we hypothesize that the effective dimension of the dynamics is approximately this number of species and resources ‘jumping’ between high- and low-abundance states. The intuition behind this phenomenon is that the hypothesis that dynamics of the aMCRM in the dynamic phase are dominated by the species and resources that are transitioning between high- and low-abundance states [26]. We hope to explore this connection further in future work.

Appendix D: Simulation and numerical methods

1. Numerical integration

Numerical integration of the differential equations is performed using the `Tsit5` solver from the `DifferentialEquations.jl` package [33] in the *Julia* programming language [34]. `Tsit5` is the Tsitouras 5/4 Runge–Kutta method, a 5th order Runge–Kutta method with an embedded 4th order method for error estimation and step size control [35]. The solver is configured to use a relative tolerance of 10^{-14} and an absolute tolerance of 10^{-14}. The aMCRM differential equations are also effectively integrated using the `VCABM` solver which an adaptive order adaptive time Adam–Moulton method [33, 36]. When using `VCABM`, the solver is configured to use a relative tolerance of 10^{-11} and an absolute tolerance of 10^{-11}. The `VCABM` is often faster than `Tsit5` for the aMCRM differential equations, but is less stable for some parameter values, particularly in the dynamic phase with low ρ and high σ. Therefore, in nearly all cases, especially those where the parameter space is explored, `Tsit5` is used.

2. Surviving/depletion cutoffs, numerical instability, and small immigration

Simulating ecological dynamics often involves including very small immigration rates, λ_N, λ_R for species and resources, respectively, to numerically regularize dynamics and ensure an uninvadable steady state is approached in simulations. Furthermore, the inclusion of small immigration ensures that chaotic fluctuations in the dynamics do not drive species or resources to extinction, killing chaos due to finite size effects. The equations used for numerical simulations are,

$$\frac{\mathrm{d}N_i}{\mathrm{d}t} = N_i\left(\sum_{\alpha=1}^{M} c_{i\alpha}R_\alpha - m_i\right) + \lambda_N, \tag{D1}$$

$$\frac{\mathrm{d}R_\alpha}{\mathrm{d}t} = R_\alpha\left(K_\alpha - R_\alpha\right) - \sum_{i=1}^{S} N_i e_{i\alpha}R_\alpha + \lambda_R. \tag{D2}$$

In all our simulations, we take $\lambda_N, \lambda_R = 10^{-10}$. Including this small level of immigration regularizes the dynamics in that the simulated steady-state abundances of species and resources now have a clear gap between the abundances of those that are surviving and those that are extinct, as shown in Fig. D14. This gap is used to define a cutoff to identify fractions of surviving species and resources. Including this small level of immigration additionally stabilizes the numerical integrator, leading to fewer simulations that report errors due to numerical instability. The effects of immigration on dynamics in the random Generalized Lotka–Volterra model is discussed in detail in [26]; we expect that many of the intuitions presented there apply to the aMCRM as well.

3. Numerically solving the cavity self-consistency equations and calculating phase boundaries

In order to solve the self-consistency equations for the cavity method, we the use nonlinear least squares method. We define the objective function to be the sum of the squares of the differences between the left and right-hand sides of the self-consistency equations (A39, A40, A44, A45, A46, A47, A42, A41):

$$L(\phi_R, \phi_N, \langle N\rangle, \langle R\rangle, q_N, q_R, \chi, \nu) = (\text{LHS} - \text{RHS of Eq. (A39)})^2 + \cdots + (\text{LHS} - \text{RHS of Eq. (A41)})^2. \tag{D3}$$

When the self-consistency equations are solved, the objective function is zero. The objective is minimized using adaptive differential evolution solver, implemented as
`BBO_adaptive_de_rand_1_bin_radiuslimited` in the *Julia* package `BlackBoxOptim.jl` [37].

There are choices of parameters $\mu_c, \mu_e, \sigma_c, \sigma_e, K, \sigma_K, m, \sigma_m, \rho$ for which the objective function does not reach zero. In Fig. D15, we plot the objective function values for various choices of ρ and $\sigma = \sigma_e = \sigma_c$; the other parameters for this figure are those used in Fig. 3. We can see that the objective function values are very small for ρ near one and suddenly jumps to a much larger value for ρ near zero. The cause of this increase in the objective function is the term (LHS − RHS of Eq. (A41)) in the objective function. Observe that as $\chi \to 0$, $-\nu \to \infty$, which leads to the objective function value blowing up. In order to find the boundary at which the objective function blows up, we simply add a term to the objective function that is zero when $\chi = 0$ and allow one model parameter (here, ρ) to vary:

$$\begin{aligned} L^{\mathrm{CSI}}(\phi_R, \phi_N, \langle N\rangle, \langle R\rangle, q_N, q_R, \chi, \nu, \rho^{\mathrm{CSI}}) =& L(\phi_R, \phi_N, \langle N\rangle, \langle R\rangle, q_N, q_R, \chi, \nu, \rho^{\mathrm{CSI}}) \\ &+ (\chi - 0)^2. \end{aligned} \tag{D4}$$

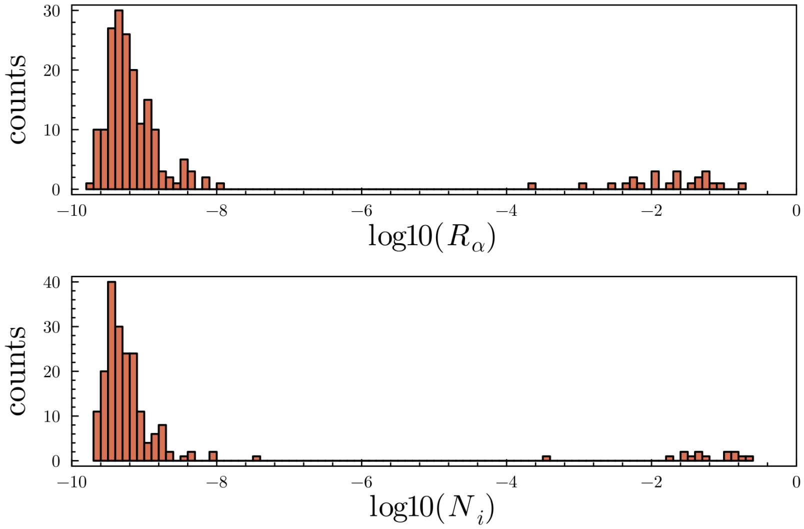

FIG. D14. Log histogram of the species and resource abundances at steady state for a system with $M, S = 256$ and $\rho = 0.9$. There is a clear gap in abundance between species/resources that are surviving and those that are extinct. The immigration rate used is $\lambda = 10^{-10}$.

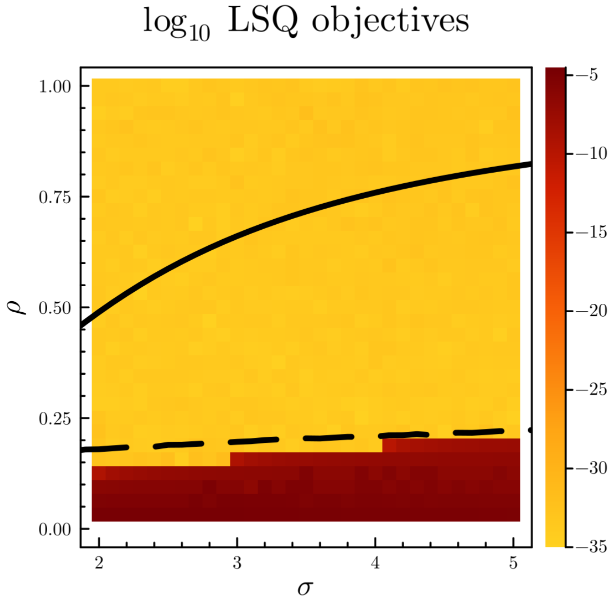

FIG. D15. Least-squares objectives for solving the cavity self-consistency equations. The solid curve is the instability boundary, and the dashed curve is the infeasibility boundary. The region in which the objective function is very small is the region in which the cavity self-consistency equations are solved; the region in which the objective function is large represents invalid solutions which are not included in plots. The parameters used are those used in Fig. 3(a).

Similarly, to find the instability boundary, we add a term to the objective function corresponding to the instability condition (Eq. (B18)):

$$L^\star(\phi_R, \phi_N, \langle N\rangle, \langle R\rangle, q_N, q_R, \chi, \nu, \rho^\star) = L(\phi_R, \phi_N, \langle N\rangle, \langle R\rangle, q_N, q_R, \chi, \nu, \rho^\star) + (\text{LHS} - \text{RHS of Eq. (B18)})^2. \tag{D5}$$

Minimizing these objective functions while allowing one additional model parameter to vary gives the infeasibility and instability boundaries shown in all figures in this paper.

Appendix E: Finite size scaling analysis

In order to understand whether the aMCRM achieves a steady state in the thermodynamic limit, we must understand how finite system size and a finite simulation time impact simulation results. To assess the existence and dynamical nature of a steady state in a simulation, we numerically calculate a time to steady state (TtSS). After running a simulation, we iterate through the time series and find the time at which the mean absolute value of the derivatives last dips below a threshold, 10^{-10}. The tolerance of the solver (`Tsit5` [33, 35]) is 10^{-14}, so this threshold is well above the numerical error of the simulation. Because we simulate to a finite time $T_{\max} = 2^{16} + 10^4 = 75536$, it is not possible to distinguish between a system that will reach a steady state at some time beyond $T_{\max}$ and a system that will never reach a steady state. Furthermore, when performing these simulations, especially with large system sizes, errors occasionally occur in the solver, and the simulation is aborted; while we do not present finite-size scaling results in regions where the simulations are frequently aborted, a robust analysis should account for this. Here, we develop a custom maximum likelihood estimation (MLE) technique to fit the distributions TtSS's at different system sizes, taking into account these two situations. We then examine the scaling of the resulting fit parameters to determine their behavior in the thermodynamic limit.

1. Modeling simulation outcomes

To construct a MLE model, we first must model the statistical process which determines each possible outcome of a simulation. We observe that simulation outcomes fall into three categories:

(i) The simulation reaches steady state within the maximum simulation time $T_{\max}$.

(ii) The simulation does not reach steady state within $T_{\max}$.

(iii) The solver encounters an error and the simulation aborts.

First, we represent the probability of a simulation encountering an error as p_{err}. Next, we consider whether a simulation could possibly reach a steady state if given enough simulation time. We represent the probability of a simulation reach steady state in the infinite time limit as ϕ_{SS}. Finally, we define the probability density function $p_{\text{SS}}(t)$ of TtSS's for those simulations that can reach steady state when given enough time.

Using these definitions, we can now construct the probabilities for each of the cases defined above. Let T be a random variable representing the observed outcome of a simulation with possible values $T \in [0, T_{\max}] \cup \{\texttt{noSS}, \texttt{error}\}$, where T takes a finite value in the interval $[0, T_{\max}]$ for case (i), or the special values `noSS` or `error` in cases (ii) and (iii), respectively.

To model case (i), we note that it results in an outcome represented by a continuous numerical value, while cases (ii) and (iii) represent categorical outcomes. For ease of derivation, we also convert T to a categorical outcome in the prior case by artificially breaking our simulation interval into small discrete time steps of size Δt. Later, we will take the limit $\Delta t \to 0$, removing the dependency of our model on this step size. The probability of a simulation completing successfully and reaching a steady state in the time interval $t < T < t + \Delta t$ with $T < T_{\max}$ is approximately

$$\mathrm{P}(t < T \leq t + \Delta t \mid T < T_{\max}) \approx (1 - p_{\text{err}})\phi_{\text{SS}} \Delta t \, p_{\text{SS}}(t). \tag{E1}$$

For case (ii), simulations do not encounter an error, but do not reach steady state within $T_{\max}$. We must consider two separate possibilities: a simulation may never reach steady state, even if $T_{\max} \to \infty$, or would reach steady-state after further simulation, $T > T_{\max}$. In the first case, the probability is simply $(1 - p_{\text{err}})(1 - \phi_{\text{SS}})$. If a simulation would eventually have reached steady-state if we had continued simulating, we must use our probability density function $p_{\text{SS}}(t)$. However, we do not know exactly when the simulation would have finished, so we must sum across all discrete time intervals greater than $T_{\max}$. The total probability for case (ii) is then

$$\begin{aligned} \mathrm{P}(\texttt{noSS}) &\approx (1 - p_{\text{err}})(1 - \phi_{\text{SS}}) + (1 - p_{\text{err}})\phi_{\text{SS}} \sum_{n=0}^{\infty} \Delta t \, p_{\text{SS}}(T_{\max} + n\Delta t) \\ &\approx (1 - p_{\text{err}})(1 - \phi_{\text{SS}} F_{\text{SS}}(T_{\max})), \end{aligned} \tag{E2}$$

where we have taken the limit $\Delta t \to 0$ and simplified, defining $F_{\text{SS}}(T) = \int_0^T dt p_{\text{SS}}(t)$ as the cumulative distribution function of $p_{\text{SS}}(t)$.

Finally, for case (iii), the probability is simply

$$\mathrm{P}(\texttt{error}) = p_{\text{err}}. \tag{E3}$$

2. Likelihood function

We now use the outcome probabilities in the previous section to construct a likelihood function to fit the data. We are given N (independent) simulations resulting in observed TtSSs $\{T_1, \cdots, T_N\}$ which may take on values $T_i \in [0, T_{\max}] \cup \{\texttt{noSS}, \texttt{error}\}$. Our fit parameters θ include p_{err}, ϕ_{SS}, along with the parameters that define the probability density function $p_{\text{SS}}(t)$. Defining the likelihood of the data given the parameters θ as $\mathrm{P}(\{T_i\}_{i=1}^N|\theta)$, the MLE estimate of the parameters is found by minimizing the negative log-likelihood,

$$\theta^\star = \operatorname*{argmax}_\theta \mathrm{P}(\{T_i\}_{i=1}^N|\theta) = -\operatorname*{argmin}_\theta \frac{1}{N}\log \mathrm{P}(\{T_i\}_{i=1}^N|\theta). \tag{E4}$$

Using our outcome probabilities, the negative log-likelihood is then

$$\begin{aligned}
\mathcal{L}(\{T_i\}_{i=1}^N;\theta) &= -\frac{1}{N}\log \mathrm{P}(\{T_i\}_{i=1}^N|\theta) = -\frac{1}{N}\log\prod_{i=1}^N \mathrm{P}(T_i|\theta) = -\frac{1}{N}\sum_{i=1}^N \log \mathrm{P}(T_i|\theta) \\
&= -\frac{1}{N}\sum_{i=1:T_i<T_{\max}}^N \log\left[(1-p_{\text{err}})\phi_{\text{SS}}\Delta t\, p_{\text{SS}}(T_i)\right] \\
&\quad -\frac{1}{N}\sum_{i=1:T_i=\texttt{noSS}}^N \log\left[(1-p_{\text{err}})(1-\phi_{\text{SS}}F_{\text{SS}}(T_{\max}))\right] - \frac{1}{N}\sum_{i=1:T_i=\texttt{noSS}}^N \log p_{\text{err}}.
\end{aligned} \tag{E5}$$

Next, we define f_{err} as the fraction of simulations that encounter errors, and the f_{SS} as the fraction of simulations that did not encounter errors and reached a steady state within time $T_{\max}$. Using these definitions, we get

$$\begin{aligned}
\mathcal{L}(\{T_i\}_{i=1}^N;\theta) &= -\frac{1}{N}\sum_{i=1:T_i<T_{\max}}^N \log p_{\text{SS}}(T_i) - f_{\text{SS}}\log\left[(1-p_{\text{err}})\phi_{\text{SS}}\Delta t\right] \\
&\quad -(1-f_{\text{err}}-f_{\text{SS}})\log\left[(1-p_{\text{err}})(1-\phi_{\text{SS}}F_{\text{SS}}(T_{\max}))\right] - f_{\text{err}}\log p_{\text{err}} \\
&= -\frac{1}{N}\sum_{i=1:T_i<T_{\max}}^N \log p_{\text{SS}}(T_i) - (1-f_{\text{err}}-f_{\text{SS}})\log\left(1-\phi_{\text{SS}}F_{\text{SS}}(T_{\max})\right) \\
&\quad - f_{\text{SS}}\log\phi_{\text{SS}} - (1-f_{\text{err}})\log(1-p_{\text{err}}) - f_{\text{err}}\log p_{\text{err}} - f_{\text{SS}}\log\Delta t.
\end{aligned} \tag{E6}$$

We note that the last term is a constant, so we may choose to ignore it and take the limit $\Delta t \to 0$ as mentioned previously, giving us the final form for the likelihood,

$$\begin{aligned}
\mathcal{L}(\{T_i\}_{i=1}^N;\theta) &= -\frac{1}{N}\sum_{i=1:T_i<T_{\max}}^N \log p_{\text{SS}}(T_i) - (1-f_{\text{err}}-f_{\text{SS}})\log\left(1-\phi_{\text{SS}}F_{\text{SS}}(T_{\max})\right) \\
&\quad - f_{\text{SS}}\log\phi_{\text{SS}} - (1-f_{\text{err}})\log(1-p_{\text{err}}) - f_{\text{err}}\log p_{\text{err}}
\end{aligned} \tag{E7}$$

3. Fitting the model

To find the maximum likelihood estimates of the parameters, we minimize Eq. (E7) using the BFGS algorithm implemented in the *Julia* package `Optimization.jl` [38]. We found empirically that the Fréchet distribution is a good choice for $p_{\text{SS}}(t)$. The Fréchet distribution has the following forms for the cumulative distribution function and probability density function:

$$F_{\text{SS}}(t) = \begin{cases} e^{-(t/\tau)^{-\alpha}}, & t > 0, \\ 0, & t \le 0, \end{cases} \qquad p_{\text{SS}}(t) = \begin{cases} \frac{\alpha}{\tau}\left(\frac{t}{\tau}\right)^{-\alpha-1} e^{-(t/\tau)^{-\alpha}}, & t > 0, \\ 0, & t \le 0, \end{cases} \tag{E8}$$

where τ is the timescale parameter and α is the shape parameter. The errors in the fitted parameters $\phi_{\text{SS}}, p_{\text{err}}, \tau, \alpha$ are computed by bootstrapping the data. That is, we randomly sample N data points from the simulation data set with replacement and fit the model to the sampled data repeatedly, providing us with an empirical distribution for each fit parameter.

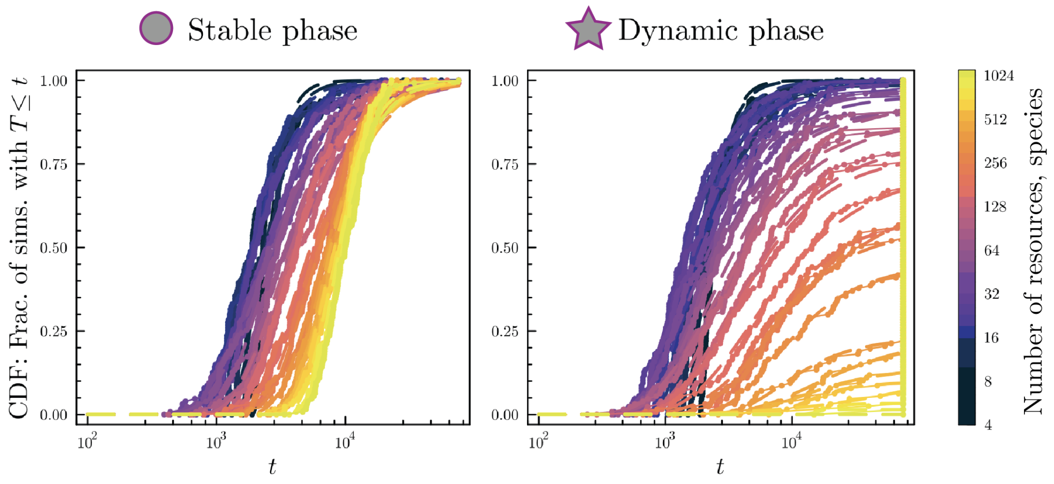

FIG. E16. Empirical cumulative distribution functions of T, the time to steady state, for simulations of different system sizes in the stable phase (left) and the dynamic phase (right). The maximum likelihood estimate distributions as described in SI section E for the time to steady state for the various system sizes are shown with dashed lines. The maximum value shown on the horizontal axis is the maximum time simulated, $T_{\max} = 2^{16} + 10^4 = 75536$.

4. Scaling of parameters with system size and discussion

The simulated data for different system sizes in the stable and dynamic phases can be visualized by analyzing the empirical cumulative distribution functions (CDFs) of the observed times to steady state called $T_{\rm SS}$ above. In Fig. E16, we show the CDFs for the stable and dynamic phases for $4 \leq M, S \leq 1024$ along with the CDFs for the fitted distributions. A clear difference is apparent between the stable and dynamic phases, and the fitted distributions match the simulated data well. A scaling collapse of the CDF based on the fitted distributions is shown in Fig. E17.

The fit parameters for the stable and dynamic phases are shown in Fig. E18. In the stable phase, the estimated probability of reaching steady state, $\phi_{\rm SS}$ remains approximately equal to 1 for all system sizes, and the estimated timescale τ asymptotically increases with system size as a power law. In the dynamic phase, $\phi_{\rm SS}$ decreases with system size towards 0, and τ asymptotically increases with system size also as a power law, but with a larger exponent. In both phases, α approaches 1 asymptotically from above.

We provide fits of $\phi_{\rm SS}$ as a function of system size M using curves of the form $\phi_{\rm SS}(M) = \phi_{\max} - \Delta(1 - \exp(-(M/\xi)^\kappa))$. For the dynamic phase $\phi_{\max} = 1.0$, $\Delta = 1.0$, $\xi = 278.5$, and $\kappa = 1.5$; for the stable phase, $\phi_{\max} = 1.0, \Delta = 0.0$, κ remains approximately equal to the value at which it is initialized in the optimization algorithm, and ξ approaches the maximum value set in the optimization algorithm.

We also provide fits of the timescale τ as a function of system size M using power laws of the form $\tau(M) = bM^a$. Curves are fit for data $M, S \geq 2^{9.5}$. In the stable phase, $a = 0.4$ and $b = 580$; in the dynamic phase, $a = 0.9$ and $b = 70$.

This finite size scaling is run in the range $512 \leq M, S \leq 1024$ but is not shown in Fig. E18 because solver errors are occasionally present. More significantly, as very few simulations reach steady state in the dynamic phase when the system size is sufficiently large, the MLE procedure cannot clearly distinguish between cases where τ is very large or $\phi_{\rm SS}$ is very small; this ambiguity can be seen more clearly in Fig. E16. With the prior knowledge of the finite size scaling as shown in Fig. E18 where the MLE procedure succeeds and there are no solver errors, we conclude that at these larger system sizes, it is both the case that τ grows large (in polynomial order of system size) and $\phi_{\rm SS}$ approaches either zero or one. That fact both the timescale τ diverges and $\phi_{\rm SS}$ approaches either zero at large system size leads us to conclude that in the thermodynamic limit, all systems in the stable phase eventually reach steady state for long enough times, while in the dynamic phase, no systems reach steady state.

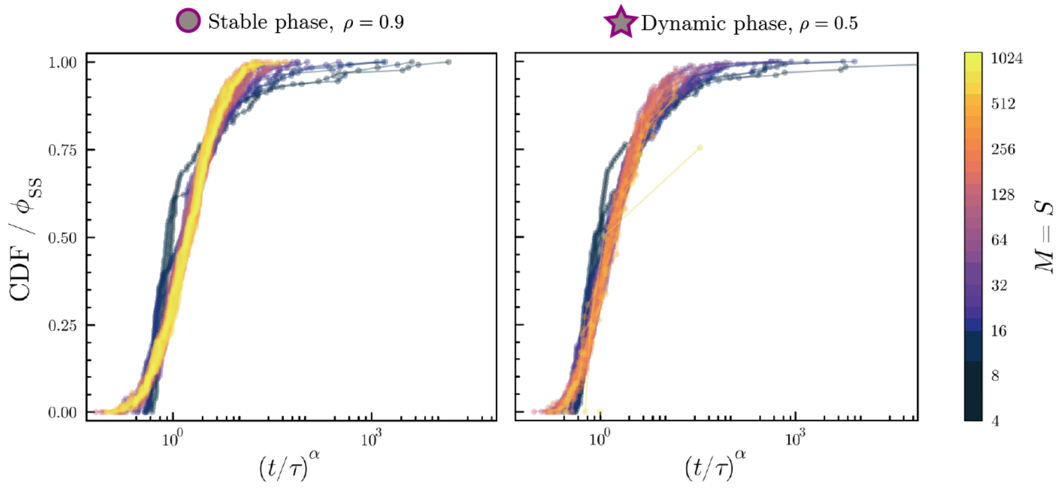

FIG. E17. Scaling collapse of TtSS CDFs. CDFs of observed TtSSs are scaled by the MLE-fitted value of ϕ_{SS} where the horizontal axis is scaled by the MLE-fitted values of τ, α. Note that the curves collapse onto each other in both phases for system sizes that are moderately large $M, S \gtrapprox 2^{4.5}$. This indicates that we have successfully identified the asymptotic scaling of the form of the TtSS distribution with system size.

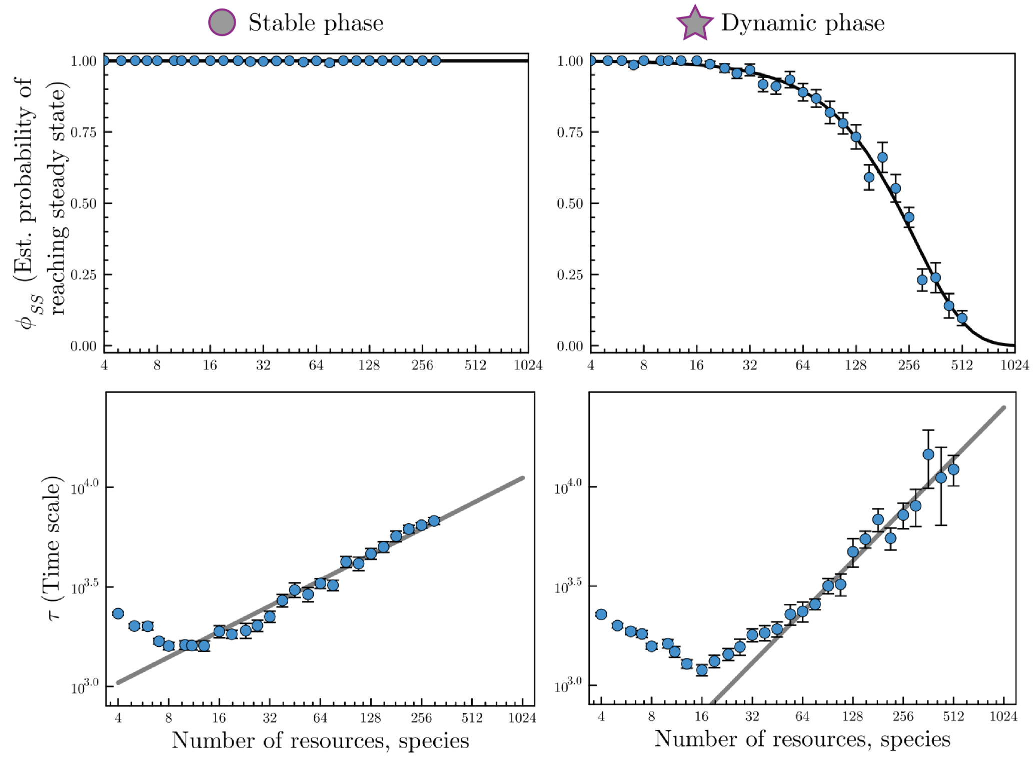

FIG. E18. Impact of finite size effects on the dynamics of the aMCRM. Rows correspond to different parameters describing dynamics: the estimated probability a simulation of a given system size will reach steady state (top) and the timescale of the time to steady state of simulations of a given system size that do reach steady state (bottom). Columns correspond to simulation data from different phases: the stable phase (left) and the dynamic phase (right); the parameters corresponding these simulations are marked in the phase diagram in Fig. 3 with a circle and star, respectively. The probability of reaching steady state in the stable phase remains at 1 for all system sizes, while the probability of reaching steady state in the unstable phase decreases to zero with increasing system size, indicating that stable dynamics are not observed within the dynamic phase in the thermodynamic limit. The timescale of the time to steady state increases with system size in both the stable and dynamic phases.

Appendix F: Additional simulations

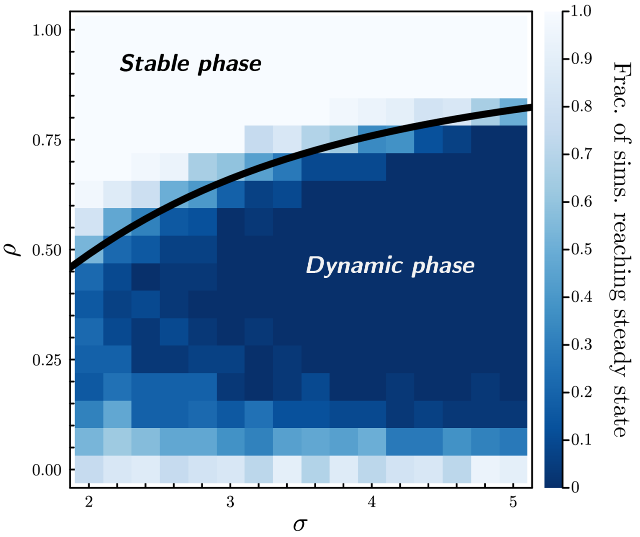

FIG. F19. Heatmap of the fraction of simulations at steady state when the sampling distributions are uniform. Parameters are the same as in Fig. 3(a). Because the calculations are performed in the thermodynamic limit, the phase boundary is agnostic to the choice of sampling distribution. Further details about sampling distribution and parameters are in appendix G.

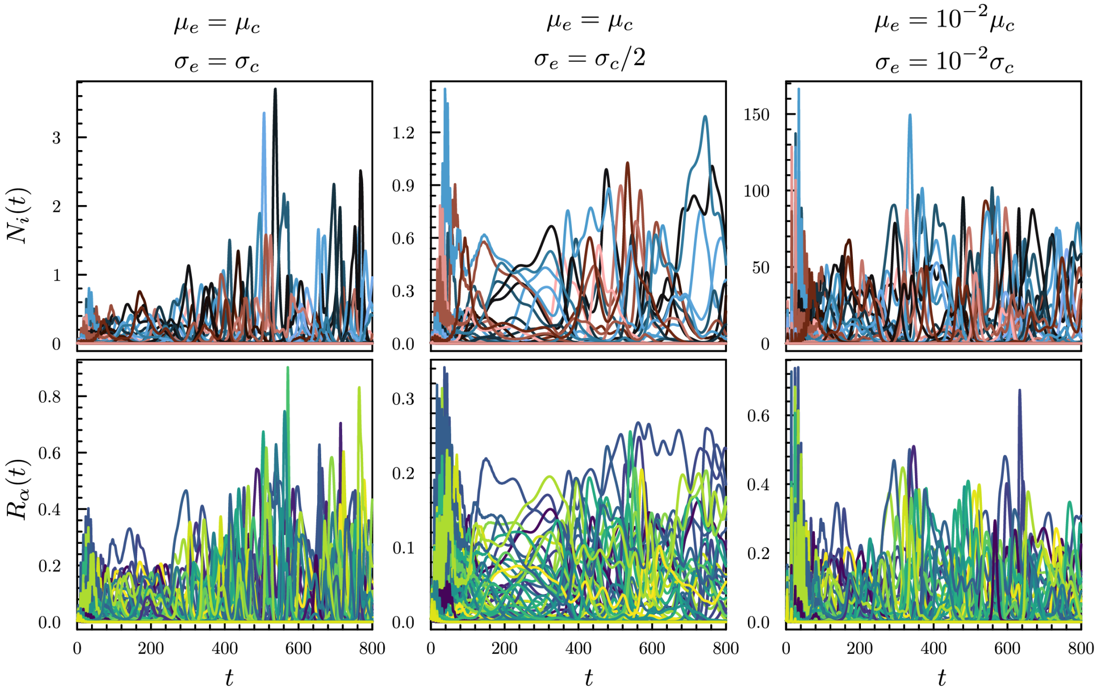

FIG. F20. Example simulations of the aMCRM with (left column) $\mu_e = \mu_c$, $\sigma_e = \sigma_c$; (center column) $\mu_e = \mu_c$, $\sigma_e = \sigma_c/2$; and (right column) $\mu_e = 10^{-2}\mu_c$, $\sigma_e = 10^{-2}\sigma_c$. The sampled random matrices and parameters are the same in each of the cases, as is the coloring of the species and resources. The chosen values of μ_c, σ_c, and ρ correspond to the gray star in Fig. 3(a).

Appendix G: Parameters in figures

Figure 1 (schematic) details

Species drawings are traces of images generated using Adobe Firefly Generative AI. Prompts: "simple 2D flat unshaded vector graphics of microbes," "simple 2D flat digital art sketches of bacteria and microbes in icon-style with no shading."

Figure 2 (example dynamics) simulation parameters

Both simulations have random variables $d_{i\alpha}, x_{i\alpha}, K_\alpha, m_i$ drawn from standard normal distributions. The parameters are,

$$K = 1, \quad \sigma_K = 0.1, \quad m = 1, \quad \sigma_m = 0.1, \quad \mu_c = 200, \quad \sigma_c = 3.5, \quad \mu_e = 200, \quad \sigma_e = 3.5, \quad \lambda_N = \lambda_R = 10^{-10}, \tag{G1}$$

with $M = S = 256$ resources and species. For dynamics in the stable phase, $\rho = 0.9$, and for the dynamics in the unstable phase, $\rho = 0.5$. Between the two simulations, the model parameters $(\delta K_\alpha, \delta m_i, d_{i\alpha}, x_{i\alpha})$ are sampled once and only the level of nonreciprocity (ρ) is changed. For these choices of parameters, $\rho^\star = 0.72$. The initial conditions are $N_i(0) = 1/S$ and $R_\alpha(0) = 1/M$. Numerical integration is performed using a 'fourth-order, five-stage explicit Runge-Kutta method with embedded error estimator of Tsitouras' implemented as `Tsit5` in the *Julia* `DifferentialEquations.jl` package [33, 35]

Figure 3 (phase diagram) simulation parameters

Each point in the heatmap represents an average over 32 simulations where the parameters were independently sampled from normal distributions: $d_{i\alpha}, x_{i\alpha}, K_\alpha, m_i \sim \mathcal{N}(0, 1)$. The distribution parameters are,

$$K = 1, \quad \sigma_K = 0.1, \quad m = 1, \quad \sigma_m = 0.1, \quad \mu_c = 200, \quad \mu_e = 200, \quad \lambda_N = \lambda_R = 10^{-10}, \tag{G2}$$

with $M = S = 256$ resources and species. The parameters σ_c and σ_e are taken to be the same: $\sigma \equiv \sigma_c = \sigma_e$. The dashed gray line represents a slice through the parameter space with the above parameters fixed and $\sigma = 3.5$ where ρ varies from 0 to 1. The gray star represents the parameters $\sigma = 3.5$ and $\rho = 0.5$; the gray circle represents the parameters $\sigma = 3.5$ and $\rho = 0.9$. The values of parameters $\mu_c, \mu_e, \sigma_c, \sigma_e$ are chosen so that when $M = S = 256$, $\langle c_{i\alpha}\rangle, \langle e_{i\alpha}\rangle = O(1)$ and $\mathrm{Var}[c_{i\alpha}], \mathrm{Var}[e_{i\alpha}] = O(1)^2$; this abates some issues involving solver errors and instabilities. The initial conditions are $N_i(0) = 1/S$ and $R_\alpha(0) = 1/M$. Numerical integration is performed using a 'fourth-order, five-stage explicit Runge-Kutta method with embedded error estimator of Tsitouras' implemented as `Tsit5` in the *Julia* `DifferentialEquations.jl` package [33, 35]. The simulations are run until 5×10^4 time units, and a simulation is considered to have reached steady state if the average absolute value of all derivatives is less than 10^{-7} within the last 0.05×10^4 time units.

The instability phase boundary (solid black) is found by solving the cavity self-consistency equations (see SI section A and Eq. (B18) simultaneously using nonlinear least squares, as described in SI section D 3).

Figure 4 (Lyapunov dot plot and diverging trajectories) simulations details

(a) Details on the calculation and analysis of Lyapunov exponents are discussed in appendix C 1. Parameters correspond to a slice of the phase diagram shown in Fig. 3(a) along the dashed gray line.

(b) Simulation parameters correspond to the gray star ($\sigma = 3.5$, $\rho = 0.5$) in Fig. 3(a). The initial conditions for the trajectory plotted in (solid) red is the state given after evolving the system for 5×10^3 time units from initial conditions, $N_i(0) = 1/S$, $R_\alpha(0) = 1/M$. The initial conditions for the trajectory plotted in (dotted) blue are generated by selecting 8 species with abundance greater than 10^{-3} at random and perturbing their abundances by a random amount drawn from a uniform distribution $U([-10^{-4}, 10^{-4}])$; the highlighted species is not one of these 8 species. The Lyapunov exponent is computed using the 'H2' method of Geist [39–42] for the trajectory plotted in red; see SI section C 1 for details.

Figure A5 (cavity distribution comparison) simulation parameters

$$K = 1, \quad \sigma_K = 0.1, \quad m = 1, \quad \sigma_m = 0.1, \quad \mu_c = 8, \quad \sigma_c = 1, \quad \mu_e = 8, \quad \sigma_e = 1, \quad \rho = 0.9 \tag{G3}$$

$$\phi_N = 0.318,\ \phi_R = 0.729,\ \nu = -0.858,\ \chi = 0.412,\ \langle N \rangle = 0.105,\ \langle R \rangle = 0.114,\ \langle N^2 \rangle = 0.0575,\ \langle R^2 \rangle = 0.0258. \tag{G4}$$

The uninvadable steady state is found using an iterative constrained optimization method described in [16]. Cavity parameters are found by solving the self-consistency equations using non-linear least squares as described in appendix D 3.

Figure F19 (phase diagram with uniform sampling) simulation parameters

All parameters are identical to those in Fig. 3(a) except that the random matrices and vectors are sampled as:

$$d_{i\alpha}, x_{i\alpha}, \delta K_\alpha, \delta m_i \sim \text{Uniform}([-\sqrt{3}, \sqrt{3}]). \tag{G5}$$

This choice of distributions ensures that $d_{i\alpha}, x_{i\alpha}, \delta K_\alpha, \delta m_i$ are still standard random variables and appropriate thermodynamic scaling are used to obtain cavity results that are identical to those in Fig. 3(a).
